# Protocol for EVOLVE-HBV, a study to determine the prevalence and characteristics of chronic hepatitis B virus infection in KwaZulu-Natal, South Africa, to inform interventions for diagnosis, treatment and prevention

**DOI:** 10.1177/27558428261459232

**Published:** 2026-08-14

**Authors:** Motswedi Anderson, Tongai G. Maponga, Janine Upton, Gloria Sukali, Lusanda Mazibuko, Kathy Baisley, Theresa Smit, Elizabeth Waddilove, Marion Delphin, Dickman Gareta, Nikiwe Mbatha, Landiwe Ndimande, Njabulo Dayi, Gregory Ording-Jespersen, Njabulo Myeni, Busangani Ngwenya, Nondumiso Mpanza, Katya Govender, Ngcebo Mhlongo, Guy Harling, Alasdair Leslie, Nomathamsanqa Majozi, Dumisani Siwela, Xanthe Hunt, Bongiwe Ndlovu, Lulama Mthethwa, Nokukhanya Msomi, M. Azim Ansari, Resign Gunda, Vasudevan G. Naidoo, Shaun R. G. Temmers, Thumbi Ndung’u, Maryam Shahmanesh, Mark Siedner, Emily B. Wong, Janet Seeley, Thandeka Khoza, Collins Iwuji, Philippa C. Matthews

**Affiliations:** 1 Africa Health Research Institute, KwaZulu-Natal, South Africa; 2 Botswana Harvard Health Partnership Institute, Gaborone, Botswana; 3 The Francis Crick Institute, London, UK; 4 Division of Medical Virology, Faculty of Medicine and Health Sciences, Stellenbosch University, Cape Town, South Africa; 5 National Health Laboratory Service, Tygerberg Business Unit, Cape Town, South Africa; 6 Division of Infection and Immunity, University College London, London, UK; 7 London School of Hygiene and Tropical Medicine, London, UK; 8 University of KwaZulu-Natal, Durban, South Africa; 9 Mortimer Market Centre, University College London Institute for Global Health, London, UK; 10 MRC/Wits Rural Public Health & Health Transitions Research Unit (Agincourt), University of the Witwatersrand, Johannesburg, South Africa; 11 Hlabisa Hospital, Somkhele, South Africa; 12 National Health Laboratory Service, Albert Luthuli Hospital, Durban, South Africa; 13 Nuffield Department of Medicine, University of Oxford, Oxford, UK; 14 Inkosi Albert Luthuli Central Hospital, Durban, South Africa; 15 Ragon Institute of MGH, MIT and Harvard, Cambridge, MA, USA; 16 HIV Pathogenesis Programme (HPP), The Doris Duke Medical Research Institute, University of KwaZulu-Natal, Durban, South Africa; 17 Department of Infectious Diseases, University College London Hospital, UK; 18 Division of Infectious Diseases, Massachusetts General Hospital, Boston, MA, USA; 19 Division of Infectious Diseases, Department of Medicine, University of Alabama at Birmingham, AL, USA; 20 Department of Global Health and Development, London School of Hygiene and Tropical Medicine, UK; 21 Department of Global Health & Infection, Brighton and Sussex Medical School, University of Sussex, UK; Current affiliation: Lulama Mthethwa and Nokukhanya Msomi are also affiliated with University of KwaZulu-Natal, Durban, South Africa

**Keywords:** HBV, hepatitis B, South Africa, infection, serology, vaccine, exposure, prevalence, epidemiology, community engagement, mental health, implementation, elimination, protocol

## Abstract

**Background:**

Hepatitis B virus (HBV) infection is endemic in many African populations, but has been neglected by funding, education, clinical infrastructure and research. High-profile international targets have been set for HBV elimination by 2030.

**Objectives:**

We here present the protocol for EVOLVE-HBV, an interdisciplinary study based at the Africa Health Research Institute aiming to quantify and characterize HBV infection in the KwaZulu-Natal (KZN) province, South Africa. The study aims to develop insights into HBV by assessing population knowledge, epidemiology, clinical features and laboratory characteristics, with the overall aim of enhancing local care pathways.

**Design:**

Study phase I is a retrospective, observational cross-sectional assessment of HBV sero-epidemiology. Phase II incorporates the prospective recruitment of adults living with HBV and HBV-negative controls from the same community. Study dates are 1 August 2023 to 31 July 2027.

**Methods:**

The study is supported by an active programme of community engagement. Phase I: banked samples are tested for HBV serum markers in a clinical diagnostic laboratory and analysed to generate weighted estimates of HBV infection, exposure and immunity. Phase II: data are collected through questionnaire, clinical assessment, biometric data, imaging using Fibroscan™ and laboratory assays including measurement of biomarkers, antiviral drug concentrations and HBV whole genome sequencing. Social scientists collect qualitative data regarding beliefs, experiences and needs of the community. Data across all themes inform a detailed picture of the sociodemographic, clinical and molecular characteristics of HBV infection.

**Ethics:**

EVOLVE-HBV is approved by the University of KZN Biomedical Research Ethics Committee in South Africa (ref. 00004495/2022), and University College London (UCL) ethics committee in the United Kingdom (ref. 23221/001).

**Discussion:**

This protocol describes a study that will inform insights into local HBV epidemiology, determine the need for scale-up of vaccination, screening and treatment, inform local policy, education and service-development and provide an evidence-based foundation for future clinical practice and translational research. Collectively, this work aims to inform improvements in clinical and public health strategies to support progress towards 2030 targets.

## Plain language summary

Protocol for EVOLVE-HBV, a study to investigate hepatitis B virus (HBV) infection in South Africa, to improve diagnosis, treatment and prevention

Hepatitis B Virus (HBV) can cause long-term infection of the liver, which may lead to liver scarring (cirrhosis) and liver cancer. Worldwide, there are goals for countries to eliminate HBV infection as a threat to their populations by the year 2030. However, we are currently off track in progress. HBV is common in many African populations, and the majority of new infections occur in Africa, but in these high-risk settings, there is often low awareness, pathways for medical care have not been well developed, and there are many gaps where more research is needed. In this paper, we present the design for a study called EVOLVE-HBV, based at the Africa Health Research Institute in KwaZulu Natal, South Africa between 1 August 2023 - 31 July 2027. We set out to study HBV infection in a rural South African population, aiming to investigate local knowledge, assess the extent of HBV infection, and describe the associated liver disease in people living with the infection, working closely with the local community. The study is divided into two parts. First, we use stored blood samples to test them for evidence of HBV infection, which helps us to estimate the number of people affected in this region. Second, we invite people living with HBV infection to attend a clinic assessment where they will complete a questionnaire, be assessed by a nurse or doctor and provide more blood samples. These samples will be used for laboratory studies to help us develop better understanding of how HBV causes liver disease, and to identify people at the highest risk of serious complications. Community engagement will inform insights into awareness, needs and expectations of the local population. Overall, the study aims to create new evidence which will help deliver better education and care for people living with HBV, will support governments in planning health services and will help drive new insights into the best monitoring and treatment, to support progress towards elimination targets.

## Introduction and rationale

Hepatitis B virus (HBV) infection is a major global public health challenge, causing over a million deaths worldwide each year.^1^ Of the 254 million people estimated to be living with chronic HBV infection worldwide, 60–80 million are in the World Health Organization (WHO) African region. However, the clinical and public health threats associated with HBV infection have been neglected^2^; <10% of people living with HBV (PLWHB) are aware of their status, <4% of those meeting treatment criteria are on therapy^3^ and data representing African populations are limited.^4^ South Africa does not have a uniformly established clinical pipeline for HBV evaluation, monitoring and treatment, and there are significant regional disparities.^5^ To meet ambitious targets for the elimination of HBV as a public health threat by 2030,^6^ rapid progress in prevention, diagnosis and treatment are required.

HBV can be suppressed with nucleos/tide analogue antiviral agents, most commonly tenofovir, given in the form of tenofovir disoproxil fumarate (TDF). This treatment lowers the risk of disease in individuals and brings population-level benefits by reducing transmission.^7^ TDF is on the WHO essential medicines list, and tenofovir is commonly part of first-line antiretroviral therapy (ART) regimens for human immunodeficiency virus (HIV), potentially creating opportunities for shared pathways, infrastructure and resources for HIV and HBV. Indeed, adults living with HBV/HIV in some settings may have better outcomes compared to those with HBV-monoinfection,^8^ likely reflecting the benefits of early and consistent therapy. HBV guidelines published by the WHO in 2024 simplify and broaden treatment eligibility criteria, providing opportunities to treat more PLWHB and advocating for decentralized services.^9^ As HBV cure efforts advance, so too does the pipeline of drugs from clinical trials to deployment. By developing a clear picture of PLWHB in different settings, along with pathways to care, foundations can be set to attract industry for clinical trials of new agents.

However, complex treatment challenges remain, including gaps in education, access and affordability.^10–12^ Although the genetic barrier to HBV resistance is high,^13^ TDF resistance mutations have been reported^14^; while currently not deemed a threat to treatment success at a population level, more work is needed to understand the prevalence, clinical impact and public health implications of drug resistance.^15^

A preventive HBV vaccine has been deployed in South Africa since 1995, but the conventional infant dosing regimen (starting at 6 weeks of age) is too late to prevent vertical transmission, and birth dose vaccine has only recently been introduced nationally,^16^ with gaps in implementation. More data are needed to describe vaccine coverage, to identify groups who may be at high risk of either missing vaccinations, or in whom protective titres of vaccine-mediated antibodies are not present despite vaccination.

This article presents a protocol for the EVOLVE-HBV (Evaluation Of Vukuzazi LiVEr disease – Hepatitis B, hereafter referred to as ‘EVOLVE’) study conducted in South Africa at the Africa Health Research Institute (AHRI). EVOLVE leverages off the existing ‘Vukuzazi’ programme in KwaZulu-Natal (KZN),^17^ using an interdisciplinary approach to tackle the pressing challenge of HBV infection. The study sets out to map the prevalence, clinical and demographic characteristics, and to describe laboratory features of HBV in this setting, aiming to generate a robust evidence base to inform education, screening, linkage to secondary care, treatment and prevention strategies.

## Ethics and timeline

### Governance and approvals

EVOLVE is conducted in accordance with the Declaration of Helsinki, the principles of Good Clinical Practice,^18^ and the ethics guidelines of the Department of Health of the Republic of South Africa. Ethical approval for Vukuzazi was previously obtained from the Ethics Committees of the University of KZN (BE560/17), London School of Hygiene & Tropical Medicine (#14722), the Partners Institutional Review Board (2018P001802), and from the University of Alabama at Birmingham (#300007237).

The AHRI Executive Committee, the AHRI Community Advisory Board (CAB), the Vukuzazi steering committee and the local Department of Health all provided approval for EVOLVE, which was then approved by the University of KZN (UKZN) Biomedical Research Ethics Committee (BREC) (ref. 00004495/2022) and University College London (UCL) ethics committee in the UK (ref. 23221/001 EVOLVE-HBV). The study is sponsored by AHRI. The AHRI population intervention programme includes the use of routine health data collected through the Health and Demographic Surveillance System (HDSS) with analysis approval from UKZN BREC (BREC; ref. BE290/16). A SPIRIT statement to support protocol design^19^ is available online (https://doi.org/10.25418/crick.29900738).

### Study timeline and context

EVOLVE started in August 2023, to run over 4 years. This published protocol represents a condensed version of the full protocol submitted for ethics review, which was most recently approved with amendments in September 2024 (protocol version 5). The study is ongoing at the time of publishing this protocol. We have altered the order, with light text editing to convert the document into a format suitable for publication. While the content of this manuscript adheres to the ethically approved protocol, we note that this document should not replace the study protocol for the purposes of day-to-day project methodology and governance.

## Aims and objectives

The overall aim of EVOLVE is to develop insights into HBV by assessing population knowledge, epidemiology, clinical features and laboratory characteristics to enhance local care pathways. Primary and secondary/exploratory study aims and key anticipated outcomes are presented in Table 1.

**Table 1.** Primary and secondary aims and projected outputs of the EVOLVE study.

Study domain	Specific aim(s)	Key outcome(s)
Primary outcomes		
Study phase I (retrospective epidemiology study)	To describe HBV epidemiology in rural KwaZulu-Natal, quantifying prevalence of active infection, previous exposure and vaccine-mediated protection in the general population.	Quantify population prevalence of HBsAg, anti-HBc and anti-HBs, corrected by weighting for the local population characteristics and presented with 95% confidence intervals around each estimate.
Study phase II (prospective clinical study)	To describe the characteristics of adults living with HBV infection and compare liver disease burden with a matched HBV-negative comparison group.	Describe distribution of ALT and fibrosis scores and report liver health in a cross-sectional cohort of adults living with and without HBV infection.
Laboratory study: quantification of drug concentrations	To quantify TFV levels in the population and relate these to control of HIV and HBV viraemia.	Determine the relationship between TFV concentrations and HIV/HBV viral load suppression.
Laboratory study: HBV sequence data	To generate HBV sequence data to determine circulating genotypes and recombinants, identify clinically relevant polymorphisms (including drug-resistance mutations) and reconstruct viral phylogeny.	Report HBV genotypes; identify and describe the prevalence of putative resistance-associated mutations and other polymorphisms of clinical significance; describe viral phylogeny.
Public health outcomes	To make recommendations on optimal clinical care pathways for people living with HBV.	Develop recommendations for evidence-based care pathways in collaboration with the local community, healthcare providers and the Department of Health.
Secondary/Exploratory outcomes		
ALT testing using POC	To validate a POC ALT test against a quantitative laboratory-based assay.	Determine metrics for ALT POC assay (sensitivity and specificity for pre-specified ALT thresholds).
Analysis of host genetic data	To generate host genetic data (focusing on HLA class I genes) to explore the impact of host genetics on HBV disease phenotypes and outcomes.	Describe associations between HLA alleles and HBV clinical and laboratory outcomes.
Description of humoral antibody responses	To determine vaccine-mediated antibody titres and describe antibody-mediated effector function.	Inform insights into the extent to which anti-HBs is protective.
Characterization of OBI	To estimate the prevalence and describe the molecular characteristics of OBI.	Report OBI prevalence, ±describe genotypes and mutations in occult infection.
Estimation of HCV and HDV prevalence	To determine the prevalence and molecular characteristics of HCV and HDV infection.	Estimate HCV and HDV prevalence, report genotypes ± polymorphisms of clinical significance.
Exploration of community experiences and education	To develop exploratory insights into local education, beliefs, behaviours, barriers to HBV care and the mental health impacts of HBV, and to use these insights to improve awareness.	Description of knowledge and attitudes, care-seeking behaviours, mental health indicators; development of educational materials.

ALT: alanine aminotransferase; HBV: hepatitis B virus; HCV: hepatitis C virus; HDV: hepatitis D virus; HLA: human leucocyte antigen; OBI: occult HBV infection; POC: point-of-care; TFV: tenofovir; HBsAg: hepatitis B surface antigen; anti-HBc: hepatitis B core antibodies; anti-HBs: HBsAg antibodies.

Primary aims are the priority goals to be addressed in this study, divided into epidemiology, clinical, laboratory and public health domains. Secondary aims are generally regarded as exploratory and descriptive in nature, aiming to generate preliminary data and hypotheses for future studies.

The project is divided into ‘phase I’ (a seroepidemiology survey undertaken retrospectively from banked samples) and ‘phase II’ (based on prospective recruitment and clinical review), supported by exploratory laboratory and social sciences studies and aiming to conclude with a combined appraisal of all the data to draw into policy recommendations.

## Methods

### Study population and setting

EVOLVE is established through the Vukuzazi framework (isiZulu for ‘wake up and know yourself’), situated in the uMkhanyakude district of rural KZN.^17^ Vukuzazi was established to investigate the overlapping health challenges of HIV, tuberculosis and non-communicable disease, offering participation to all resident members of the area aged ⩾15 years between May 2018 and March 2020. EVOLVE leveraged off Vukuzazi’s infrastructure, data and biobank, expertise in HIV diagnosis and management and interdisciplinary approaches to research and healthcare.

### Community engagement

The CAB is part of AHRI’s Public Engagement strategy, acting as a bridge between the community and the research institute. The CAB comprises elected community representatives to ensure that research activities are discussed with – and informed by – local communities, and that research provides opportunities for community education and partnerships. This approach improves the likelihood that recommendations and outputs can be translated to real-world implementation. The CAB raised questions and provided feedback to support EVOLVE study design prior to ethics submission, and a CAB meeting in January 2024 further informed development of research tools, including a Topic Guide to inform interviews and focus groups.^20,21^ Ongoing CAB engagement events are scheduled throughout the study. The AHRI Public Engagement and Social Science teams provide expertise in assimilating and interpreting data from the community and stakeholders.^22^

### HBV seroepidemiology study (study phase I)

EVOLVE draws retrospectively on banked Vukuzazi samples, to screen a subsection of the population for serological evidence of HBV infection, exposure and vaccination, stratifying according to age, sex and HIV status of the participant at the time of collection (Figure 1). Analysis draws on 2200 selected samples, to represent population groups born in different ‘epochs’: pre-vaccine roll-out (born before 1995), peri-vaccine roll-out (born 1995–2000) and post vaccine-roll out (born 2000–2005). Samples are selected based on availability and/or volume. Where a choice of available samples in a given category is present, a random selection is made using a computer algorithm. Each sample is tested for the following HBV markers:

i. Hepatitis B surface antigen (HBsAg) as a marker of HBV infection;
ii. Hepatitis B core antibodies (anti-HBc) as a marker of exposure to infection;
iii. HBsAg antibodies (anti-HBs) as a marker of exposure and/or vaccination. Antibody titres are quantified to determine the proportion of those positive who exceed thresholds commonly used to infer immune-mediated protection (most commonly 10 or 100 IU/ml).

**Figure 1.**
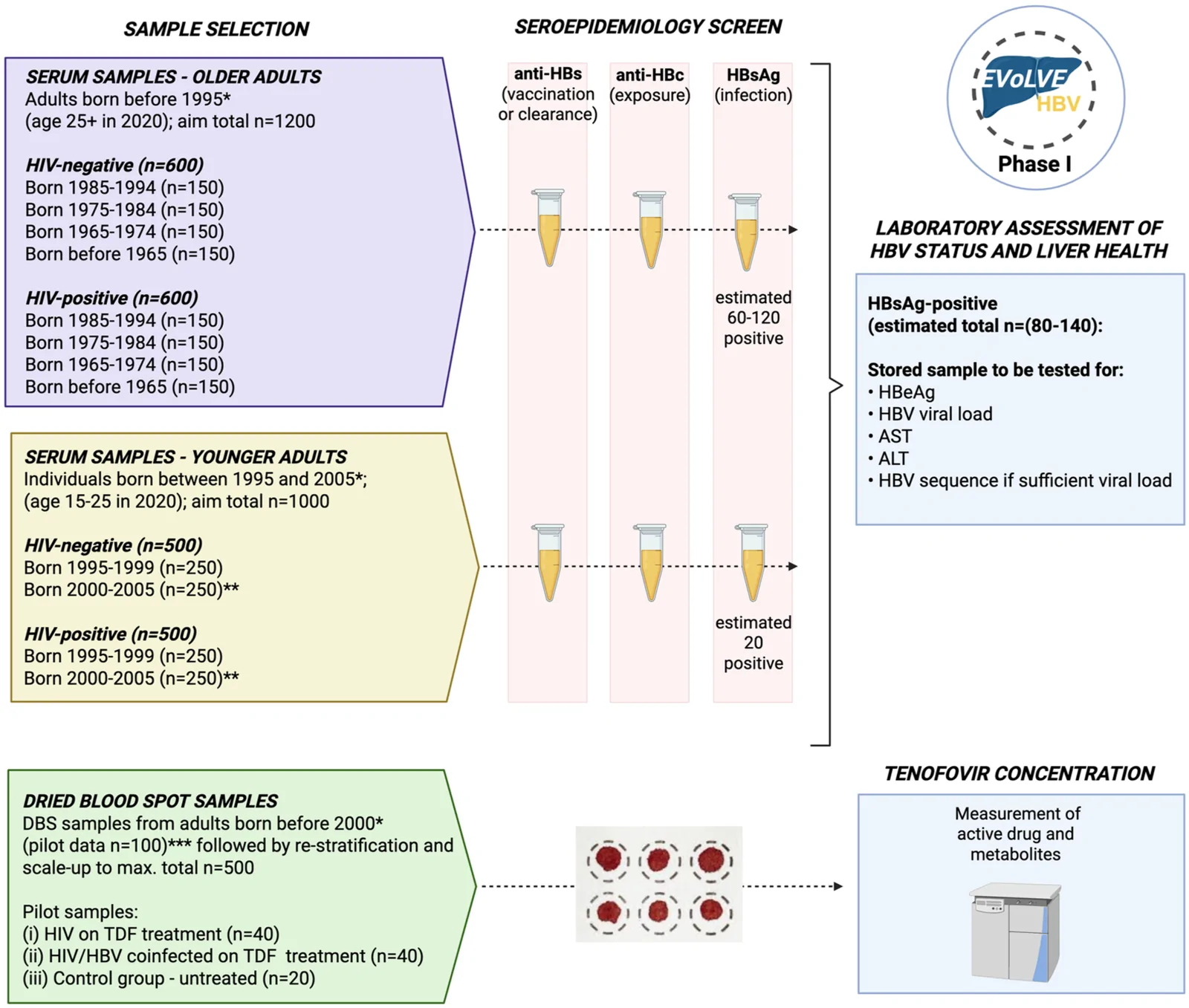
Schematic approach to EVOLVE-HBV study design for phase I, showing selection of samples for HBV serological testing and measurement of drug concentrations. Source: Figure created by EVOLVE-HBV study team in https://BioRender.com with a license to publish. HBV: hepatitis B virus; ALT: alanine aminotransferase; HBeAg: hepatitis B e antigen; AST: aspartate aminotransferase; TDF: tenofovir disoproxil fumarate; HIV: human immunodeficiency virus; HBsAg: hepatitis B surface antigen; anti-HBs: hepatitis B surface antibodies; anti-HBc: hepatitis B core antibodies. Based on HBV prevalence estimates (4%–8%) before the study started, with differences between age strata, we estimated 60–120 HBsAg positive cases among older adults and 20 positive in younger adults. *Age categories were defined based on hepatitis B vaccine introduction periods. **Sample numbers in this group are provisional and may be adjusted depending on availability, particularly for participants born between 2000 and 2005. ***Dried blood spot samples will be preferentially selected from participants included in the sero-epidemiological screening cohort.

### Prospective clinical review (study phase II)

The prospective component of the study is represented in Figure 2. The primary aim of phase II is to describe the demographic, clinical and laboratory characteristics of PLWHB in our study setting, and to compare these characteristics to a matched HBV-negative population (Table 1).

**Figure 2.**
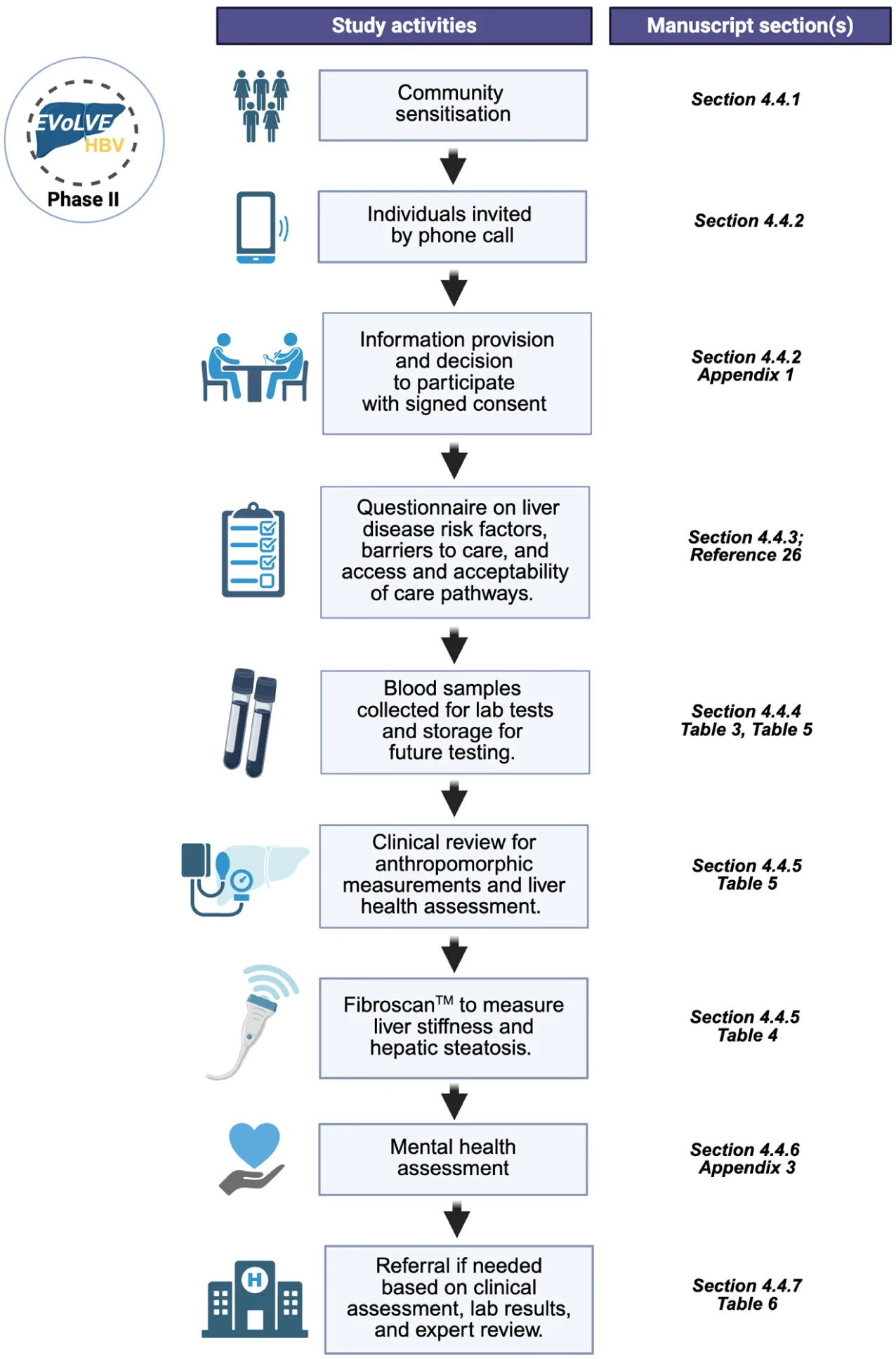
Schematic illustration of clinic assessment for participants in the EVOLVE-HBV study phase II. The relevant section of the protocol is referenced for each step of the pathway. Source: Figure created by EVOLVE-HBV study team in https://BioRender.com with a licence to publish.

#### Community sensitization prior to recruitment

Prior to prospective study initiation, community engagements through the CAB and community leaders present and explain the study and invite questions and discussion. Leaflets, radio broadcasts and roadshows provide information and raise awareness of HBV within the community. Materials^20^ and details of engagement events^23,24^ are available online.

#### Recruitment and consent

Based on the results of the retrospective screening, eligible participants are selected (Table 2) and contacted in person or by telephone to participate in study phase II. Recruitment focuses on two groups:

**Table 2.** Inclusion and exclusion criteria for participation in phase II of the EVOLVE-HBV study.

Inclusion criteria	Exclusion criteria
• Adults (age 18+) • Able to provide valid informed consent • Not pregnant • HBV positive group: test positive for HBsAg and/or HBV DNA at the time of data collection and on at least one previous test (on a stored sample) • HBV negative group: test negative for HBsAg. Anti-HBc may be positive or negative.	• Age <18 • Do not wish to participate or unable to provide valid informed consent • Pregnant • Previous sample tested HBsAg-positive but negative on reassessment (eligible to participate as a control but not a case)

HBV: hepatitis B virus; HBsAg: hepatitis B surface antigen; anti-HBc: hepatitis B core antibodies; DNA: deoxyribonucleic acid.

1. PLWHB: All individuals whose samples have tested positive for HBsAg in study phase I. Based on estimated HBV prevalence of between 4% and 8% in adults, at the outset of the study we anticipated identifying a cohort of 80–160 individuals.
2. HBV-negative group: A control group (matched 2:1 to HBsAg-positive cases) who are HBsAg-negative and household contacts of PLWHB (subject to agreement of the index participant), and/or from the general community. Household members who test HBsAg-positive will be offered the opportunity to participate in the study as part of the HBV cohort. If we do not identify/recruit sufficient negative controls through this approach, we will expand recruitment by identifying HBsAg-negative adults from the general population who are matched by age and sex to our cases, from phase I. Matched controls are selected at random in cases in which there is a choice of potential participants.

Data collection from HBV-negative participants allows an assessment of all-cause liver disease in the wider community. This provides preliminary data regarding the prevalence and severity of liver disease caused by non-HBV aetiology (e.g. alcoholic liver disease, metabolic liver disease, inflammatory or fibrotic liver disease caused by exposure to other infections, drugs and toxins). Definitions and criteria for metabolic-dysfunction associated steatotic liver disease have been published in 2023,^25^ since the original protocol development and will be applied retrospectively to the EVOLVE dataset.

A trained member of the study team explains the research and offers prospective participants an opportunity to attend clinic review. Participant identity is first verified by cross-checking the national identification document against the corresponding electronic database entry. Supplemental information is provided (infographic, written/verbal form) to help them decide whether they would like to participate (Supplemental Appendix 1). Whilst paper copies of participant information leaflets and consent forms are available if needed, most of the study documents are online (in line with AHRI practice) and signed electronically. Any questions are addressed by a member of the study team.

#### Questionnaire

At the study visit, participants are supported to complete a self-report questionnaire; this includes questions on risk factors for liver disease, barriers to care and access and acceptability of HBV care pathways.

#### Blood collection and testing

A venous blood draw of 25–30 ml is collected for routine clinical laboratory assays (Table 3) performed by the National Health Laboratory Services (NHLS) Department of Virology at Albert Luthuli Hospital in Durban. Short-term storage of samples is undertaken at the NHLS lab at 4–8° in case repeat testing is required. Residual samples are frozen at −80° to allow for additional testing, subject to funding and ethics approvals.

**Table 3.** Sample types and volumes to be collected from EVOLVE-HBV study participants enrolled in prospective review (study phase II).

Sample type/volume	Testing
Dried blood spot (200 µl whole blood on filter paper)	Drug concentrations
Whole blood or plasma, according to the manufacturer’s instructions (blood spot)	ALT POC test (lateral flow device)
Serum separation tubes (collect 20 ml)	HBV serology (HBsAg, HBeAg, antiHBe) (3 ml)
	Liver function tests (1 ml) – (ALT, AST, GGT, ALP, BR, Alb)
	Lipid profile (500 µl)
	HbA1c (500 µl)
	HCV antibody/antigen (1 ml)
	HBV sequencing (5 ml)
	Alphafetoprotein (500 µl)
	Creatinine (1 ml)
	Stored sample for aflatoxin (500 µl)
	Residual sample to be stored (approx. 5 ml)
EDTA tubes (collect 25 ml)	HBV ± HIV viral load (3 ml)
	Full blood count ± CD4+ T cell count (2 ml)
	Whole blood for storage (3 ml)
	PBMC processing for storage (17 ml)
Urine sample (collect 2 ml)	Biochemistry and protein:creatinine ratio

AST: aspartate aminotransferase; HBsAg: hepatitis B surface antigen; HBeAg: hepatitis B e antigen; anti-HBe: hepatitis B e-antibody; ALT: alanine aminotransferase; GGT: gamma-glutamyltransferase; ALP: alkaline phosphatase, BR: bilirubin, Alb: albumin; HbA1c: haemoglobin A1c; HCV: hepatitis C virus; HBV: hepatitis B virus; HIV: human immunodeficiency virus; PBMC: peripheral blood mononuclear cells; POC: point-of-care.

Results are automatically uploaded into the study Research Electronic Data Capture (REDCap) instance, from the NHLS Laboratory Information Management System; see section ‘Data collection’ section for further details of data management. Results are reviewed by a member of the clinical or study team, and liver fibrosis scores are calculated and classified using the thresholds in Table 4. Alerts are set up for the study team triggered by any laboratory results that need priority clinical action.

**Table 4.** Calculation of scores to estimate liver fibrosis from laboratory data.

Score	Formula	Threshold/s to predict fibrosis	Sources and metrics of fibrosis threshold (derived from previous studies)
APRI^a^	((AST/ULN AST)/platelet count)x100	0.5	APRI thresholds revised by the HEPSANET consortium^26^
			Updated WHO guidelines use treatment threshold of 0.5^9^
		0.7^a^	Sensitivity: 77%
			Specificity: 72%
			Derived from HCV studies^27^
FIB-4	(Age in years × AST)/(platelet count × √ALT)	3.25	Specificity: 97%
			Positive predictive value: 65%
			Derived from HCV studies^28^
GPR	((GGT/ULN of GGT)/platelet count) × 100	0.32	Optimal cut-off value for predicting significant fibrosis derived from individuals with chronic HBV infection in West Africa^29^
S-index	(1000 × GGT)/(platelet count × Albumin^2^)	0.3	Specificity: 94%
			Positive predictive value: 87% Derived from individuals with chronic HBV infection in Egypt^30^

AST: aspartate aminotransferase at u/l; ALT: alanine aminotransferase at u/l; APRI: AST to platelet ratio index; GGT: gamma-glutamyltransferase at u/l; GPR: GGT to platelets ratio; HCV: hepatitis C virus; HBV: hepatitis B virus; ULN: upper limit of normal; Platelet count at 10^9^/L.

a APRI threshold of 0.7 quoted in original EVOLVE protocol but subsequently superceded by lower threshold of 0.5 due to updated evidence.

#### Clinical and liver health assessment

A clinical review is performed by a doctor at the AHRI Clinical Trials Unit to identify any signs of chronic liver disease (e.g. ascites, jaundice, hepatomegaly, spider naevi). The study procured a Fibroscanner™ (Echosens, Paris) and trained local practitioners on its use (training report available online^31^), with probe sizes M and XL. Liver health assessment involves (i) liver stiffness measurement by transient elastography, which identifies inflammation or fibrosis/cirrhosis and provides a quantitative value (in kilopascals, range 2–75 kPa); and (ii) controlled attenuation parameter (CAP) measurement, an indicator of hepatic steatosis, expressed in decibels per meter (range 100–400 dB/m; Supplemental Appendix 2: Supplemental Table 1). At least 10 valid readings are collected to generate a median and IQR. The procedure is quick (typically 5-10 minutes) and non-invasive.

#### Mental health assessment

The prevalence of psychosocial disorders in PLWHB is reported to be higher than in non-HBV populations^32–35^; we here consider reactions to a new diagnosis,^36,37^ and impact on overall wellbeing.^38^ A pilot mental health assessment is undertaken irrespective of HBV status. Standard scoring thresholds are applied to trigger alerts for severe depression, anxiety or stigma.^39–41^ Standard mental health scales (PHQ9, GAD5 and the SSCI) are used, which have been previously validated and applied in South Africa,^39–42^ and scoring thresholds used to trigger alerts where the participant reports severe depression, anxiety or stigma (Supplemental Appendix 3). Any concerns are reviewed with the clinical research team in a regular meeting and can be escalated more quickly if concerns are identified. The EVOLVE mental health protocol is available online.^43^

#### Clinical referral for additional investigation and/or specialist input

The clinical component of our study interfaces closely with delivery of healthcare interventions. The project was developed in collaboration with clinical practitioners in the AHRI clinical trials unit, in the local district hospital (Hlabisa) and in the regional urban referral centre (Albert Luthuli Hospital in Durban). While the study generates clinical data, ongoing management and treatment are outside the scope of the research, and clinical decision-making and follow-up is undertaken based on local recommendations for best practice through established care pathways.

Data for all individuals with parameters that need follow-up are discussed in an expert panel comprising clinicians trained in general medicine, infectious diseases and hepatology, with expertise in the management of HBV infection in the United Kingdom and South Africa (Supplemental Appendix 4). Meetings are convened monthly. Assessments are based on all laboratory and clinical data collected (Table 5). Cases are put forward for clinical discussion based on meeting pre-specified criteria (Table 6). At study inception, we planned that clinical decision-making would refer primarily to South African guidelines, but we have subsequently accounted for the release of new international guidelines published by the WHO.^9^ Management will be continued by existing local services. Since treatment is outside the remit of the study, application of new guidelines does not influence our research outcomes, and we recognize that some individuals living with HBV will already be taking HBV-active antiviral therapy if treated for HIV.

**Table 5.** Data collection using clinical and laboratory to assess liver disease in EVOLVE phase II.

Category	Data to be collected	Approach
Socio-demographic factors	Age at clinical review	Self-report
	Sex	Study questionnaire
	Location of residence	Existing health records
	HIV co-infection	Vukuzazi records
	Alcohol	GPS data
	Smoking	
Biometric data	Body mass index	Measurement by study team at clinic visit using approved methods for collecting anthropometric data
	Waist-hip ratio	
	Blood pressure	
Clinical factors	Drugs/medications	Study questionnaire
	History of diabetes	Existing health records
		Vukuzazi records
Viral factors	(i) HBV serology (HBsAg, anti-HBs, anti-HBc, HBeAg)	10 ml serum tube
		10 ml EDTA tube
	(ii) HBV (±HIV if co-infected) viral loads	Serum or plasma sample for HBV sequencing, HCV antibody testing; follow-up with HCV RNA test for antibody-positive samples
	(iii) CD4+ T cell count and % in individuals living with HIV coinfection.	
	(iv) Screening for HCV co-infection.	Stored sample for future HDV testing^a^
Assessment of liver disease	Liver function tests (ALT, AST, GGT, ALP, BR, Alb)	EDTA blood tube
		Serum sample for alphafetoprotein (1 ml)
	Platelet count	Residual sample stored for future measuring of aflatoxin^b^
	AFP	Fibroscanner™ (Echosens)
	Aflatoxin	
	Elastography and CAP	
Renal and metabolic disease and drug concentrations	Renal function (creatinine to be measured, from which we will calculate eGFR)	Serum sample for creatinine (1 ml)
		Dried blood spot
	Lipid profile	Urine sample (2 ml) for measurement of protein:creatinine ratio and urinary phosphate
	HbA1c	
	Tenofovir concentrations	

CAP: controlled attenuation parameter; GPS: global positioning system; HIV: human immunodeficiency; AFP: alpha fetoprotein; HbA1c: haemoglobin A1c; HBV: hepatitis B virus; HBsAg: hepatitis B surface antigen; anti-HBs: hepatitis B surface antibodies; anti-HBc: hepatitis B core antibodies; HBeAg: hepatitis B e antigen; HCV: hepatitis C virus; ALT: alanine aminotransferase; AST: Aspartate aminotransferase; GGT: gamma-glutamyltransferase; ALP: alkaline phosphatase; BR: bilirubin, Alb: albumin; HCC: hepatocellular carcinoma; EDTA: ethylene-diamine-tetra-acetic acid; RNA: ribonucleic acid; HDV: hepatitis D virus; APRI: AST to platelet ratio index; FIB-4: fibrosis-4 index.

a HDV test not available locally at the time of study initiation, but aliquots to be stored to allow potential future testing.

b Aflatoxin assays are not available locally, but aliquots to be stored to allow potential future testing.

**Table 6.** Criteria for clinical discussion of research participants in EVOLVE phase II with infectious diseases/hepatology team.

Classification	Clinical criteria	Lab/imaging criteria^a^
Group 1	Acute liver failure, decompensated cirrhosis, advanced fibrosis or cirrhosis – suggested by clinical history or examination findings of jaundice, ascites, history of gastrointestinal bleeding within the past 12 months, unexplained peripheral oedema, unexplained severe bruising	Detectable serum HBV DNA (irrespective of fibroscan and ALT)
Group 2	May be symptomatic or asymptomatic	Fibrosis score F2 with HBV DNA >2000 IU/ml and ALT > ULN
Group 3	May be symptomatic or asymptomatic	Fibrosis score F3 or F4 irrespective of HBV DNA and ALT
Group 4	May be symptomatic or asymptomatic	HBV DNA >20,000 IU/ml and ALT >2× ULN, irrespective of fibrosis score
Group 5	May be symptomatic or asymptomatic	HBeAg-positive chronic HBV infection, with HBV DNA levels >20,000 IU/ml, if >age 30 years
Group 6	At the discretion of the clinic team, for example based on potential future pregnancies, family history of liver disease, co-factors (alcohol, viral co-infection), health-care workers, elevated AFP, other clinical suspicion of liver disease or extra-hepatic manifestations of HBV infection.	Detectable serum HBV DNA (irrespective of fibroscan and ALT)
Group 7	May be symptomatic or asymptomatic	If fibroscan cannot be obtained, we will use laboratory scores based on published thresholds of APRI and FIB-4
Group 8	HCV or HDV infection	HCV antibody and HCV RNA positive, or HDV RNA positive

HBV: hepatitis B virus; DNA: deoxyribonucleic acid; ALT: alanine aminotransferase; ULN: upper limit of normal; HBeAg: hepatitis B e antigen; HCV: hepatitis C virus; AFP: alpha fetoprotein; RNA: ribonucleic acid; HDV: hepatitis D virus; APRI: aspartate aminotransferase to platelet ratio index; FIB-4: fibrosis-4 index.

a Defined thresholds have been set with reference to national and international guidelines, including South African HBV recommendations^44^ and guidelines that were active at the time of protocol development (European Association for the Study of the Liver (EASL^45^) and the UK National Institute for Clinical Excellence (NICE).^46^ The protocol states that specific thresholds applied can be changed to maintain congruence with clinical guidelines, recognizing the likelihood of new guidelines being published during the study period (e.g. updated EASL guidelines published in 2025^47^).

### Laboratory characterization of HBV infection

Prospective samples collected in phase II (in some cases supported by samples stored historically through the Vukuzazi programme) are used for laboratory characterization of HBV. This protocol provides a brief methods overview; additional supporting details for laboratory studies can be found in the Supplemental Appendix 5. On publication of individual results, detailed methods will be presented, ensuring all laboratory work and data analysis are reproducible. A standard operating procedure used by our laboratory teams is available online.^48^

#### Measurement of drug concentrations

Concentrations of free TFV and its metabolite (tenofovir diphosphate) are measured from serum/plasma and dried blood spots, respectively. A method for their quantification using high-pressure liquid chromatography coupled to tandem mass spectrometry has been developed and validated at the Pharmacology Core facility at AHRI (Supplemental Appendix 5.1).^49^

#### Pathogen sequencing

We aim to derive HBV whole genome sequences, where HBV DNA is above the threshold for sequencing, using banked and new serum samples from PLWHB, using the published ‘HEP-TILE’ method^50^ (Supplemental Appendix 5.2: Supplemental Table 2).

#### Alanine aminotransferase measurement

Immunoassay point of care (PoC) lateral flow tests for alanine aminotransferase (ALT) are being developed (e.g. Nanjing DeShi Diagnostic Technology Co. Ltd^51^), but these require further validation especially in settings lacking reliable access to laboratory testing. These tests are loaded with a blood or plasma sample and can be read at 30 minutes with a visual read based on a defined threshold (e.g. ALT >40 IU/ml; Supplemental Appendix 5.3). Testing will be subject to procurement of PoC kits.

#### Host genetics

All participants are asked for consent for generation and exploratory analysis of genetic data. In some cases, this has already been confirmed as a result of their participation in the Vukuzazi programme; this consent is reviewed with the participant (Supplemental Appendix 5.4).

#### Humoral immune responses

Serum and peripheral blood mononuclear cells are collected for further exploration of immune responses. We will explore vaccine-mediated antibody titres and antibody-mediated effector function, to inform insights into the extent to which anti-HBs is protective (based on thresholds of 10, 100 or 1000 IU/ml), and to support the design of future larger future studies (Supplemental Appendix 5.5: Supplemental Table 3).

#### Occult HBV infection

Residual samples from both phases I and II of the project that have tested HBsAg-negative are stored for potential future exploratory analyses for occult HBV infection, using screening of individual or pooled serum samples for HBV DNA (Supplemental Appendix 5.6).

#### Hepatitis C and D virus coinfection

Hepatitis C virus (HCV) antibodies are checked and positive results followed up with RNA PCR at NHLS on Abbott Alinity assay and Cepheid Xpert platforms as part of a comprehensive assessment of liver health. As an exploratory objective, all (or a subset) of samples are tested for hepatitis D virus (HDV) RNA at NHLS labs. This is not currently a routine test available through NHLS, but standard and validated assays may be purchased. Any cases in which HDV RNA is detected are referred to the hepatology team for review. Further details are in Supplemental Appendix 5.7.

### Social sciences

Qualitative data from the local population are collected to evaluate how HBV is understood and experienced (including transmission beliefs and exploratory analyses of stigma), to identify barriers and challenges, and to obtain community feedback to inform the development of an accessible and acceptable HBV clinical care pathway. Our methods involve the following approaches:

(a) Dialogue with clinical and research teams directly involved in the EVOLVE-HBV project

Feedback meetings with clinical teams are conducted on up to four occasions spanning the duration of phase II, to troubleshoot recruitment, data collection or clinical difficulties, provide support for staff, collate feedback around the acceptability of research and care pathways, share ideas pertaining to service development, education and support for PLWHB. The findings from these touchpoints are then disseminated to stakeholders, including medical officers, healthcare workers and representatives from local hepatology services and members of the local community.

(b) Data collection through questionnaires

A small number of questions in the participant questionnaire address expectations for clinical services and barriers to care, as an approach to collection of quantitative data.

(c) In depth interviews

We reviewed, piloted and refined an interview schedule during January 2024 to develop a Topic Guide.^20^ This guide is used in subsequent in depth interviews (IDIs) with up to 30 people who are and who are not living with HBV infection, drawn from the Vukuzazi population to gain insights into the awareness, beliefs, challenges and suggestions for change in the local population.

(d) Focus group discussions

Information from the CAB engagement, healthcare worker feedback and the IDIs will guide discussions in up to four focus group discussions (FGD) with local leaders, members of the Vukuzazi population, the public engagement team, social scientists and representatives of the clinical research team. Sampling to saturation determines the cut-off points for IDIs and FGDs. These numbers are based on extensive experience of qualitative data collection in the uMkhanyakude community, and on standard qualitative practices.

## Data collection

### Electronic study records

Data from clinical reviews, questionnaires and laboratory tests are captured electronically using REDCap. Participants are allocated a unique personal identifier (re-using Vukuzazi ID where relevant). Information collected is in the data dictionary (Supplemental Appendix 6: Supplemental Table 4). During the study, we undertake periodic evaluation and management checks to assess data completeness, rate of recruitment and data consistency (quality control check undertaken weekly and analysis of 10% of all records monthly).

### Extraction of routine electronic data

Detailed data are already stored for Vukuzazi participants, including comorbidity, drug therapy, demographics, biometric data, laboratory and imaging data. A geographical information system holds details of geolocation and residence. Where possible, HIV treatment records are accessed from TIER.Net for people living with HIV, including unique identification number, age, sex, date of ART initiation, viral load (VL) results and dates, CD4 count results and dates, ART regimen, status of the individual (in care, lost to follow-up, deceased) and dates of clinic appointments. This currently applies for data collected up to May 2020. Later data will be added if linkage with TIER.Net is resumed.

### Data management responsibilities

The study principal investigator (PI) and local AHRI PI are data controllers, and the Executive Director of AHRI is data controller for Vukuzazi. The data custodian at AHRI is assigned to the data management team with oversight from the data controllers. AHRI, The Francis Crick Institute (UK), UCL (UK) and collaborators at the University of Oxford (UK) are data processors. UK sites only process anonymized data. It is the responsibility of the data custodian to ensure that data access is consistent with terms of the ethical approvals. Data are shared subject to relevant contracts and agreements between parties.

AHRI has a waiver through protocol BE290/16 not to seek individual written consent for linkage of routine service delivery data with the platform data because (a) the research involves no more than minimal risks to the participants, (b) this combined dataset is restricted to anonymized data and (c) analytical results are reported at population or group level and not at individual level.

### Data processing and storage

Data are processed and stored on servers under the physical control of AHRI, subject to industry-standard data integrity and user authentication for access control. Data are replicated on at least a daily basis for secure offsite storage, and transactional logs are backed up regularly to enable recovery of data in the event of equipment failure. Data flow is shown in Figure 3. Management of personally identifiable information, data audit, archiving and data sharing between research sites are undertaken per local AHRI policy (Supplemental Appendix 7).

**Figure 3.**
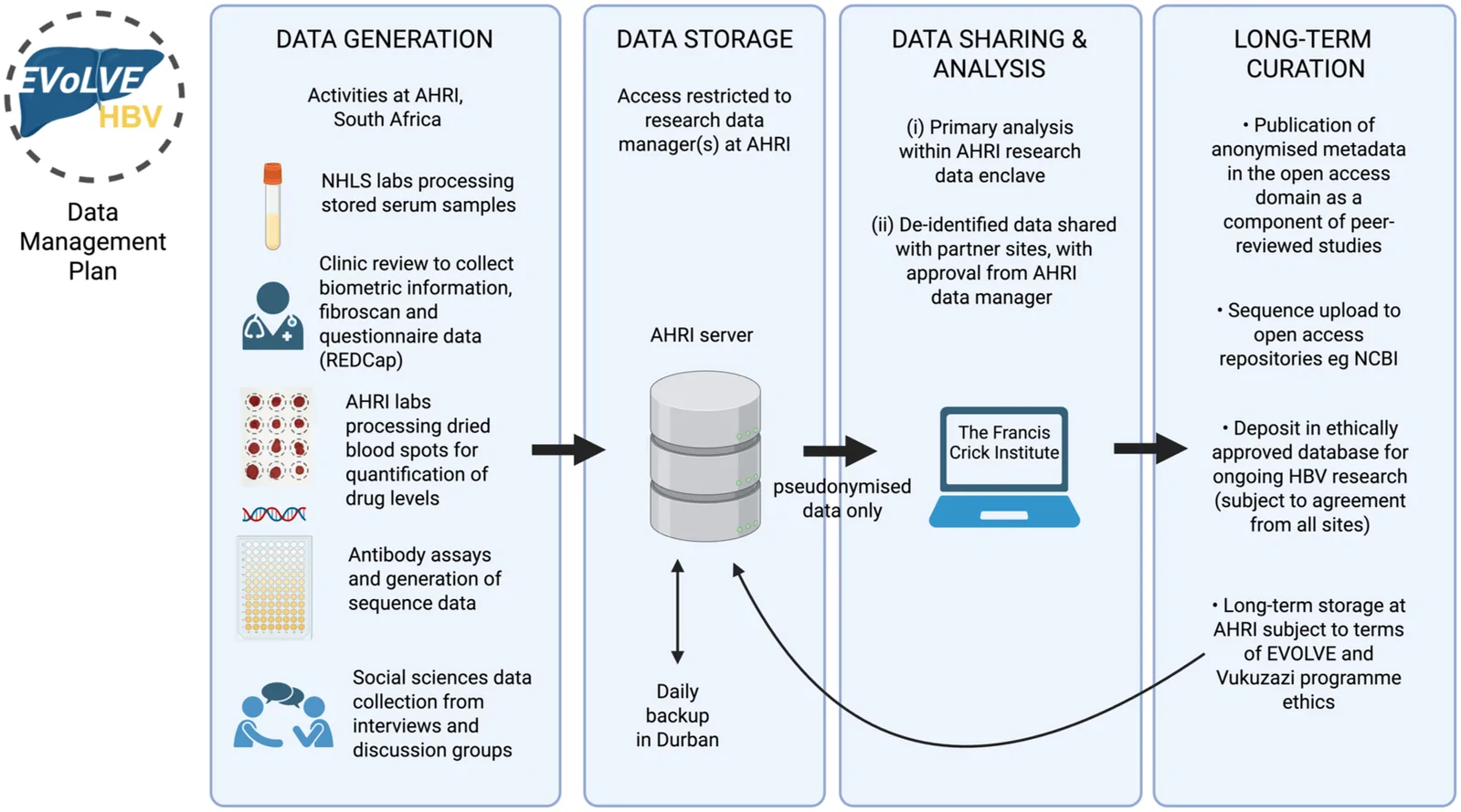
Flow diagram to show data flow and data management plan for the EVOLVE-HBV project. Source: Figure created by EVOLVE-HBV study team in https://BioRender.com with a licence to publish. NHLS: National Health Laboratory Services; AHRI: Africa Health Research Institute; REDCAP: Research Electronic Data Capture; HBV: hepatitis B virus; NCBI: National Center for Biotechnology Information.

## Statistics and data analysis

### Study phase I: Sample size and analysis

Assuming the prevalence of HBV infection in the population is 4%–8% and age-related differences, before starting the study we expected to find between 60 and 120 cases of HBV infection among older adults and 20 cases in younger adults in a sample of 2200. According to our sample size, we could estimate a prevalence of 8% with a precision of ±1.2%. For a prevalence of 4%, our precision is ±1.9%.

The data allow us to calculate the population prevalence of HBV serological markers, and to stratify these by age, sex and HIV status and by location of residence, with confidence intervals where applicable. This allows us to estimate the prevalence of HBV infection (HBsAg), vaccine-mediated immunity (anti-HBs alone) and exposure (anti-HBc). To derive population-level prevalence estimates, analyses will incorporate sampling weights to correct for differential selection probabilities within the Vukuzazi biobank. These weights are calculated as the inverse of the probability of selection across strata defined by age, sex and HIV status. To further account for variation in programme participation, additional adjustment for non-participation is undertaken using inverse probability weights based on age and sex-specific participation patterns. Confidence intervals for weighted prevalences are calculated using a logit transformation, with Taylor series linearization variance estimation.

### Study phase II: Sample size and analysis

We will generate the following descriptive outputs from prospectively collected data:

- Characteristics of liver disease among PLWHB, focusing on treatment eligibility and the proportion of untreated individuals meeting antiviral criteria.
- Hepatic fibrosis and steatosis status generated from laboratory and/or imaging data (transient elastography).
- Liver disease markers, HBV VL, renal function and alpha-fetoprotein described according to HBV status and antiviral treatment status.

Based on data on liver function tests from a population-based survey of 9000 participants in Uganda,^52^ we estimate the prevalence of abnormal liver function tests may be 5%–15%. We calculated power based on sample size, case:control ratio and prevalence of abnormal laboratory markers (Supplemental Appendix 8: Supplemental Table 5). Based on a pre-study estimate of 100 HBV cases, recruitment of two HBsAg-negative controls per case and a 10% prevalence of abnormal laboratory markers among controls, the study has ⩾80% power to detect an odds ratio of ⩾2.5 between cases and controls.

Missingness in phase II is anticipated to be low based on a prospective approach to data collection, and banked samples which can be accessed to fill gaps in laboratory data if they arise. Regular review of our REDCap data base allows us to identify gaps and re-contact participants to complete missing details. Where missingness occurs, its extent and patterns will be described. If missing data exceed minimal levels or demonstrate evidence of systematic patterns, appropriate methods such as multiple imputation will be implemented, with sensitivity analyses conducted to assess the robustness of findings. Potential selection bias arising from recruitment or non-response will be assessed by comparing key baseline characteristics (e.g. age, sex, HIV status and other available demographic variables) between participants enrolled in phase II and those eligible but not recruited from phase I. Where differences are identified, their potential impact on study findings will be evaluated through sensitivity analyses, including stratified analyses and regression-based adjustment. In addition, inverse probability weighting will be considered to account for differential participation.

A priori covariates included age, sex, HBV infection status, HIV status, alcohol use and metabolic risk factors (BMI, diabetes), given their established roles in liver injury and fibrosis progression. Regression models will be selected according to outcome type, including logistic regression for binary outcomes, linear regression for approximately normally distributed continuous outcomes, and transformed linear models for skewed continuous outcomes such as liver stiffness measures.

### Analysis of laboratory data

In this section, we present a brief analysis plan for the study’s primary aims. Additional details are provided as Supplemental Materials (Supplemental Appendix 5).

#### Analysis of drug concentrations

We will quantify concentrations of TFV and TDF-DP and use these data to determine:

- The population distribution of drug levels in males and females on TFV (± other antiretroviral drugs) with HIV and/or HBV infection;
- The relationship between drug concentrations and other individual parameters (e.g. age, sex, BMI, liver function, renal function, urinary creatinine, protein and phosphate);
- The relationship between drug concentrations and suppression of HIV and/or HBV VL;
- The relationship between drug concentrations and the presence of confirmed or putative HBV drug resistance polymorphisms.

#### Analysis of sequencing data

Our team is developing a bioinformatic pipeline for handling HBV sequence data and thus has an existing approach to analysis, with appropriate expertise to support data handling and optimization of outputs. HBV sequence outputs are presented in Supplemental Table 2. This part of the study is exploratory and descriptive and not subject to an a priori power calculation.

#### Analysis of host genetic data

We will describe the distribution of HLA alleles between groups as follows:

- HBsAg-positive (infected) versus HBsAg-positive/anti-HBc positive (exposed but not infected);
- HBeAg-positive versus HBeAg-negative;
- Presence/absence of HBV polymorphisms (e.g. CD8+ T cell escape).

We will use conditional logistic regression models, adjusted for multiple comparisons and potential confounding factors such as HIV status and CD4 cell count, and viral genotype (Supplemental Appendix 5.4). As HLA is a highly genetically diverse locus, this study is unlikely to be powered to detect significant effects, but data can be pooled with data from other sources to enhance statistical power as part of a wider effort to understand the interplay between host genetics, viral genome and clinical outcomes of HBV infection.

### Analysis of barriers to care and qualitative data

Qualitative and quantitative data collected for the social sciences component of the study will be cross-referenced and compared to build a rounded evidence base, incorporating data and methodologies from different sources to enhance the dependability and credibility of findings (Table 7).

**Table 7.** Social sciences data collection and analysis activities.

Category	Activity/approach	Details of data collection, storage and output
Data collection and analysis	Cross-reference qualitative and quantitative data; triangulate methodologies	Combine data sources to enhance dependability and credibility of findings, improving understanding of HBV.
Qualitative data collection	In-depth interviews and focus group discussions	Conducted by trained AHRI Social Science staff with informed consent, in private settings. Recordings stored securely; data anonymized and confidentiality ensured.
Data management	Secure storage and transcription	Recordings stored on password-protected devices; transcribed, translated and verified against audio. Access limited to designated study staff.
Ethical practices	Confidentiality and anonymity	Pseudonyms assigned; clear confidentiality statements at start of each session; anonymized reporting.
Community engagement	Generate advocacy and education recommendations	Use social norms approaches to explore influences on HBV testing/treatment decisions. Identify community supporters/influencers.
Planned outputs	Communication materials and engagement activities	Develop improved materials, social media content, school/youth group programmes, radio broadcasts and personal stories to encourage community support for PLWHB.
Stakeholder engagement	CAB and public meetings	Schedule meetings at 3–6, 6–9 and during year 2 to discuss care pathways and refine outputs.
Clinical care pathway	Establish referral pipeline and explore telemedicine options	Framework for routine care pathways during/beyond study. Include telemedicine where appropriate to reduce travel costs/disruption.
Collaborative partnerships	Work with other ethically approved projects	Include integrated sexual and reproductive health studies (e.g. HIV PrEP provision^50^). Operate under EVOLVE-HBV governance framework.

CAB: Community Advisory Board; HBV: hepatitis B virus; PLWHB: people living with hepatitis B virus; HIV: human immunodeficiency virus; PrEP: pre-exposure prophylaxis; AHRI: African Health Research Institute.

Analysis will be guided by topics generated by the research objectives and codes will also be drawn from the data to develop a codebook led by the AHRI Social Sciences team. A process of constant comparison and debriefing among the team will be used during coding to ensure consistency and also to facilitate the discussion and documentation of emerging patterns in the data. For the quantitative data, the team will determine the proportion of cases and controls reporting barriers to potential HBV care and acceptability of care pathways, testing for differences using *t*-tests or via regression models.

Through collaboration with other ethically approved projects, studying community-based integrated sexual reproductive health to provide HIV pre-exposure prophylaxis (PrEP) at AHRI, EVOLVE explores opportunities for decentralized care delivery.^53^

## Study management

### Study management group

The study management group includes the PI, an AHRI faculty co-lead and co-investigators leading Social Sciences and laboratory domains. A weekly team meeting is convened online throughout the period of the study including clinicians and nurses, laboratory managers and scientists, public engagement, finance and statisticians.

### Benefits of the study

For individual study participants, the study provides a detailed review of liver health which is not available through existing clinical services and provides access to information, awareness and knowledge. Through the study, we can provide simple advice and information that supports liver health (e.g. alcohol reduction, attaining a healthy weight). For PLWHB this knowledge can be empowering in managing liver health, avoiding potential transmission risks, seeking consultation if symptoms of concern arise, developing awareness in families and households, and providing access to specialist consultation if required. It is also possible that we identify cases of other chronic diseases from which participants will benefit from intervention and/or clinical referral (e.g. diabetes, hypertension).

Training healthcare staff empowers them to learn about HBV transmission, surveillance and management. We will enhance skills in the use of the Fibroscanner™ relevant not only to assessment of HBV but also to liver disease more widely. Developing insights and understanding about HBV infection in the local community will encourage discussion and awareness. Our data describing and characterizing HBV infection in the population will be essential for industry collaborations as we move into the era of cure therapies.

The datasets we will generate are unique in the level of depth, building on Vukuzazi infrastructure, adding imaging assessments (rarely available in African healthcare settings), and including HBV sequencing and drug levels. Studies of the impact of TFV are of crucial importance as the HIV treatment landscape changes (e.g. move to injectable therapy and/or dual therapy which may not include HBV-active agents). The data we generate through this programme will be informative for prevention, diagnosis, surveillance and treatment in other African settings and further afield. All clinical decision-making and patient management pathways follow standard-of-care guidelines, with care further optimized through multidisciplinary team review linking clinical data with research data.

### Risks of the study

Venepuncture (to collect up to 30 ml of blood) is routine and low-risk and will only be undertaken by trained phlebotomists using aseptic technique. However, the procedure may be uncomfortable, there is a risk of bruising, and a very small risk of local infection. We will adhere to all routine infection control precautions to reduce risks for both clinical and community-based work.

We will make a new diagnosis of chronic hepatitis B infection in some individuals, which could cause anxiety or be associated with stigma and discrimination. We will aim to manage and mitigate this with the careful provision of education and advice, with access to trained research and clinical teams. We may identify individuals (with or without HBV infection) with evidence of liver disease (or other chronic disease, such as diabetes or hypertension) who require further clinical appraisal. Among individuals who need long-term follow-up, there is a potential social or economic impact; we will provide information about options for clinical follow-up to optimize access pathways.

Household members will only be invited with the permission of the index participant who has tested positive for HBsAg, to avoid concerns around unintended disclosure of their HBV status. For household members who test negative for HBsAg, anti-HBs and anti-HBc, we will also discuss access to HBV vaccination.

It will be made known to participants that they have the right to withdraw from the study at any time, and that identifiable information will be protected such that the research team cannot link data to identifiable individuals.

### Reimbursements

Reimbursements will apply for participants attending clinical review or engaging in the Social Sciences research, to compensate for time and inconvenience. This will involve a one-off payment of R100, with additional remuneration if travel costs need to be included (proportionate to the distance travelled, up to a maximum of R800). This amount is within acceptable limits as per National Health Research Ethics Committee, and is not considered undue inducement.

### Capacity building

This project has supported recruitment of a new post-doctoral research fellow, a PhD fellowship, investment in laboratory scale-up for HBV assays and molecular work. By building an international interdisciplinary team with expertise in HBV, we develop opportunities for ongoing HBV-focused work. Purchase of a Fibroscanner™ is a significant investment in hardware, supported by staff training, with potential benefits to other clinical studies. Healthcare professionals, support workers, communities and research participants will be provided with education about HBV infection. Members of the interdisciplinary team will benefit from training and enrichment opportunities. We intend for the clinical pathways that are established as a result of this study to be a precedent for ongoing/future HBV care in this population and to provide a basis for future clinical trials.

### Dissemination

Research findings will be shared with the CAB and the district and provincial Department of Health for discussion and to inform planning of priorities and interventions. Investigators will aim to share outputs on institutional web sites, through use of pre-print publication, by publishing findings in peer-reviewed journals and presenting at national and international conferences. Study materials are being made available for reuse in an online collection.^54^

Some findings from the EVOLVE-HBV project have already been published at the time this protocol manuscript goes to press. Publications include a community dialogue study that explored local knowledge, beliefs and practices related to HBV,^22^ and a pilot investigation of HBV care pathways among individuals receiving PrEP, conducted in collaboration with partner studies.^53^

## Conclusion

HBV is a pathogen of international health importance that causes a disproportionate burden of infection and disease in the WHO African region. This project aims to create important new data to characterize HBV seroepidemiology in a South African population, and to generate sociological, clinical, laboratory and molecular insights. The project will provide a foundation for establishment of enhanced clinical care for PLWHB and for interventions to prevent new infections and reduce the risk of liver disease. Establishing local expertise in HBV alongside an evidence base for intervention will support future studies, underpinning advances towards ambitious international elimination targets for 2030.

## Supplemental Material

sj-docx-1-taj-10.1177_27558428261459232 – Supplemental material for Protocol for EVOLVE-HBV, a study to determine the prevalence and characteristics of chronic hepatitis B virus infection in KwaZulu-Natal, South Africa, to inform interventions for diagnosis, treatment and prevention Supplemental material, sj-docx-1-taj-10.1177_27558428261459232 for Protocol for EVOLVE-HBV, a study to determine the prevalence and characteristics of chronic hepatitis B virus infection in KwaZulu-Natal, South Africa, to inform interventions for diagnosis, treatment and prevention by Motswedi Anderson, Tongai G. Maponga, Janine Upton, Gloria Sukali, Lusanda Mazibuko, Kathy Baisley, Theresa Smit, Elizabeth Waddilove, Marion Delphin, Dickman Gareta, Nikiwe Mbatha, Landiwe Ndimande, Njabulo Dayi, Gregory Ording-Jespersen, Njabulo Myeni, Busangani Ngwenya, Nondumiso Mpanza, Katya Govender, Ngcebo Mhlongo, Guy Harling, Alasdair Leslie, Nomathamsanqa Majozi, Dumisani Siwela, Xanthe Hunt, Bongiwe Ndlovu, Lulama Mthethwa, Nokukhanya Msomi, M. Azim Ansari, Resign Gunda, Vasudevan G. Naidoo, Shaun R. G. Temmers, Thumbi Ndung’u, Maryam Shahmanesh, Mark Siedner, Emily B. Wong, Janet Seeley, Thandeka Khoza, Collins Iwuji and Philippa C. Matthews in Sage Open Chronic Disease

## Data Availability

Appendices are made available as a supplement to this peer-reviewed protocol. A SPIRIT checklist for protocol publication, based on standards set by EQUATOR guidelines, is available online: https://doi.org/10.25418/crick.29900738 A collection of study materials, resources, reports and outputs is available online at the following link: https://doi.org/10.6084/m9.figshare.c.7678727
