# Supplementary material for: Protocol for EVOLVE-HBV, a study to determine the prevalence and characteristics of chronic hepatitis B virus infection in KwaZulu-Natal, South Africa, to inform interventions for diagnosis, treatment and prevention
Source: Sage Open Chronic Dis. 2026 Aug 14;17:27558428261459232. doi: 10.1177/27558428261459232 (PMC13768701; doi:10.1177/27558428261459232)
Supplement: Supplementary file 1 [file sj-docx-1-taj-10.1177_27558428261459232.docx]

Supplementary material

**Protocol for ‘EVoLVE-HBV’, a study to determine the prevalence and characteristics**

**of chronic hepatitis B virus (HBV) infection in KwaZulu-Natal, South Africa,**

**to inform interventions for diagnosis, treatment and prevention**

Contents of Supplement

[**Appendix 1: Participant Information and Informed Consent**	3](#_Toc221956293)

[**Appendix 2: Analysis plan for prospectively collected clinical data (study Phase II)**	4](#_Toc221956294)

[Supplementary Table 1: Classification of liver disease using transient elastography and controlled attenuation parameter, collected by Fibroscan^TM^ (Echosens, Paris)	5](#_Toc221956295)

[**Appendix 3: Mental Health Assessment Tools**	6](#_Toc221956296)

[**Appendix 4: Study oversight and management**	7](#_Toc221956297)

[4.1 Routine meetings	7](#_Toc221956298)

[4.2 Clinical review and referrals	7](#_Toc221956299)

[**Appendix 5: Laboratory methods and analysis of laboratory data**	8](#_Toc221956300)

[5.1 Measurement of Tenofovir (TFV) concentrations	8](#_Toc221956301)

[5.2 HBV Whole Genome Sequencing (WGS)	9](#_Toc221956302)

[Supplementary Table 2: Analysis approaches for HBV sequence data	11](#_Toc221956303)

[5.3 ALT point of care testing (POCT)	12](#_Toc221956304)

[5.4 Host genetics	13](#_Toc221956305)

[5.5 Humoral immune responses	14](#_Toc221956306)

[Supplementary Table 3: Aims for studies of the humoral immune response to HBV	14](#_Toc221956307)

[5.6 Occult HBV Infection (OBI)	16](#_Toc221956308)

[5.7 HCV and HDV coinfection	17](#_Toc221956309)

[**Appendix 6: Data dictionary**	18](#_Toc221956310)

[Supplementary Table 4: Data dictionary for EVOLVE-HBV study	18](#_Toc221956311)

[**Appendix 7: Supplementary details of data management plan**	24](#_Toc221956312)

[7.1 Data storage	24](#_Toc221956313)

[7.2 Data availability	25](#_Toc221956314)

[**Appendix 8: Exemplar sample size calculation for clinical data**	26](#_Toc221956315)

[Supplementary Table 5: Anticipated power for detection of varied odds ratios (OR), according to the prevalence of an abnormal biomarker and varying numbers of HBV cases.	26](#_Toc221956316)

[**Supplementary References**	27](#_Toc221956317)

#### **Appendix 1: Participant Information and Informed Consent**

Provisional participants will be provided with verbal, written and infographic material to explain the study. For those who wish to participate, they will be asked to sign clinical study, social sciences and genetic study informed consent forms, which are provided on-line (using a study tablet) and discussed with a trained study nurse who collects electronic consent. Paper forms will be made available upon request if needed.

Participant information and consent forms explain the study aims and intended outcomes. They explain that participants can choose whether to take part, offer them the opportunity to ask further questions, and explain that they are free to withdraw from the study at any time without influencing any aspects of their healthcare provision. A list of tests performed and data collected is provided. Information forms include a list of the benefits and potential risks of taking part, and provide contact details for the team conducting the study.

Study information is explained verbally in participants’ preferred language by trained staff, with opportunities for questions and clarification. Understanding is assessed using a teach-back approach, and participants may involve a trusted support person during the consent process if desired. In addition, a simplified infographic information form is also provided to improve accessibility, especially to help explain all elements of the study to those with limited literacy; this is provided below and is also available on-line.

#### **Appendix 2: Analysis plan for prospectively collected clinical data (study Phase II)**

Based on the prospective clinical dataset generated by EVOLVE-HBV, the following outputs will be generated:

- A description of demographic, clinical features, laboratory and imaging parameters, and risk factors for liver disease in PLWHB and a control group of individuals who test HBsAg-negative.
- Calculation of laboratory fibrosis scores, Gamma-glutamyl transpeptidase to platelet ratio (GPR), Aspartate aminotransferase (AST) to Platelet Ratio Index (APRI) and FIB-4 (based on liver function tests and platelet count); application of these scores to estimate the prevalence of established liver fibrosis, and assessment of the extent to which these laboratory-derived scores correlate with liver stiffness measurement by transient elastography (**Supplementary table 1)**.
- A calculation of AST/ALT ratio (ratio >2 is associated with alcoholic liver disease), and determination of the extent to which this ratio correlates with self-reported alcohol intake.
- Among PLWHB, an assessment of eligibility for antiviral treatment, allowing us to determine the proportion of PLWHB who are currently untreated but eligible for antiviral treatment based on South African guidelines, and WHO guidelines.
- A statistical description of the distribution of HBV VL, in individuals on and off therapy.
- Assessment of associations between demographic, host and clinical factors with liver disease (based on laboratory and/or imaging scores), including HIV/HBV infection, prescribed or over-the-counter medication, metabolic disease (based on diabetes, body mass index, HbA1c, lipid profile and hepatic steatosis measured by controlled attenuation parameter), alcohol intake.
- A description of individuals with elevated Alpha-fetoprotein (AFP) as a potential marker of liver disease including hepatocellular carcinoma (these individuals will be discussed with clinical hepatology/gastroenterology to be considered for additional assessment on clinical grounds), through referral to the local district hospital and/or on to a teaching hospital centre.
- Estimation of the prevalence of chronic kidney disease, through measuring serum creatinine, calculation of estimated glomerular filtration rate (eGFR), ± urinary biomarkers.

###### Supplementary Table 1: Classification of liver disease using transient elastography and controlled attenuation parameter, collected by Fibroscan^TM^ (Echosens, Paris)

| **Liver disease** | **Threshold*** | **Classification** | **Interpretation** |
| --- | --- | --- | --- |
| **Fibrosis** (transient elastography)** | 7 kPa** | Treatment threshold | Treatment threshold published by WHO[^38^](https://paperpile.com/c/3htKyC/wpwZj) |
|  | <8 kPa | F0-F1 | No liver scarring or mild liver scarring |
|  | 8.0-9.9 kPa | F2 | Moderate liver scarring |
|  | 10.0-11.9 kPa | F3 | Severe liver scarring |
|  | ≥12 kPa | F4 | Advanced liver scarring |
|  | >12.5 kPa** | Cirrhosis | Cirrhosis threshold published by WHO[^38^](https://paperpile.com/c/3htKyC/wpwZj) |
| **Steatosis**  **(controlled attenuation parameter)** | 230 dB/m******* | S0 (Binary steatosis threshold) | A lowered threshold of 230 dB/m to define steatosis vs. non-steatosis[^39^](https://paperpile.com/c/3htKyC/GWX4b). |
|  | <238 dB/m | S0 | No fatty liver disease |
|  | 238-260 dB/m | S1 | 11-33% steatosis (mild steatosis) |
|  | 261-290 dB/m | S2 | 34-66% steatosis (moderate steatosis) |
|  | >290 dB/m | S3 | >66% steatosis (severe steatosis) |

* Specific numerical thresholds may be changed to maintain congruence with clinical guidelines which are updated over time, and to allow us to explore the influence of changing thresholds on the estimation of the prevalence of liver disease in this population.

** Thresholds included in WHO HBV guidelines published in 2024[^38^](https://paperpile.com/c/3htKyC/wpwZj); these were not included in the original EVOLVE protocol but will be applied retrospectively for analysis purposes.

*** CAP scores to grade steatosis vary, with a single threshold of 230 dB/m proposed as a cut-off to distinguish the presence/absence of steatosis.

#### **Appendix 3: Mental Health Assessment Tools**

- **Generalized Anxiety Disorder 7-item (GAD-7):** A reliable instrument, with reported 89% sensitivity and 82% specificity for the measurement of anxiety. The scale will be used to generate a classification scheme, utilising weighted responses to classify persons into categorical measures for anxiety. These measures will be used descriptively to understand the landscape of anxiety across our study population.
- **Patient Health Questionnaire-9 (PHQ-9):** PHQ-9 is a measure for depression, with a sensitivity of 88% and a specificity of 88%. The PHQ-9 is the depression module, which scores each of the 9 DSM-IV criteria as “0” (not at all) to “3” (nearly every day).
- The **SSCI-8** scale measures stigma and is composed of 8 items, each rated on a 5-point Likert scale (1 = never, 5 = always), and the total score is the sum of all item scores. The total score range from 8 to 40. Higher scores indicate a greater perception of stigma associated with the chronic illness. A higher total score (closer to 40) indicates that the individual perceives more stigma related to their chronic illness. This score does not contribute to the mental health referral pathway but will be assessed as part of the outcomes from the study.

#### **Appendix 4: Study oversight and management**

##### 4.1 Routine meetings

The study oversight is the responsibility of the PI supported by AHRI Faculty Lead. A study management group will meet regularly to review data emanating from the project and will be the main decision making body of the study. All those involved in the study team will undergo detailed briefing and education about HBV infection, and receive the full protocol and study materials. Meetings will be held with nursing and medical staff before and during the study to provide ongoing education and opportunities for discussion. Management decisions and strategy are planned with AHRI Faculty representatives including clinicians, laboratory scientists, and social scientists. A weekly team meeting is convened on-line to review progress, discuss challenges, and plan next steps.

##### 4.2 Clinical review and referrals

In any participant in whom research/clinical appraisal raises immediate concerns, same day/following day discussion will be held with the team at Hlabisa Hospital, with the potential for further discussion with (or transfer to) the hepatology team in Durban. If required on clinical grounds, we will reach out to other experts in our clinical centres. Further assessment and intervention will be done based on clinical need (outside the remit of the study). Cases will be put forward for clinical discussion based on meeting any of the criteria in **Table 6**, in an on-line meeting scheduled regularly during periods of active recruitment. Additional interim discussions can be scheduled on an *ad hoc* basis on the basis of clinical need. Contemporaneous records will be kept of cases discussed.

#### **Appendix 5: Laboratory methods and analysis of laboratory data**

##### 5.1 Measurement of Tenofovir (TFV) concentrations

TFV is phosphorylated intracellularly to form its metabolite TFV-Diphosphate (TFV-DP), which accumulates and has a 17-day half-life, providing a quantitative measure of treatment adherence. We aim to measure both free TFV (in serum or plasma) and the TFV-DP metabolite (in cellular samples such as PBMCs or dried blood spots), to determine individual and population TFV exposure, and to investigate the relationship between drug concentrations, HBV viral load (VL), and genotypic evidence of antiviral drug resistance, considering outcomes in individuals and at the population level.

We will generate pilot data from 100 samples, which will then be used to plan stratified testing of drug concentrations within an extended set of up to a total of 500 samples. Initially making use of pre-banked samples, we will measure drug concentrations to develop a picture of levels in individuals and in the population.

Selection of the first 100 to derive a preliminary data set will represent the following groups:

- Adults not on TFV based therapy (HIV/HBV negative), n=20 (negative control set).
- Adults on TFV therapy with HIV/HBV coinfection, n=up to 40 (depending on the number identified).
- Adults on TFV therapy for HIV mono-infection, n=at least 40 (to make the total sample set up to 100).

We will ideally select samples taken from adults represented in the seroepidemiology study (EVOLVE Phase I) and take a pragmatic approach to sample selection, working from the most recently stored and working back in time until we have a complete sample set. If there is a choice of available samples in a given category, we will select based on access to samples that permit VL testing, and then choose a final selection at random using a computer algorithm.

Up to an additional 400 samples will be selected after analysis of the pilot sample set of 100, to optimise precision estimates for population subgroups (for example, analysis by age, sex or other characteristics). This may include samples collected from participants enrolled in Phase II of the study, to help us include a balanced sample that includes individuals with HBV monoinfection.

Analysis will be undertaken as follows:

1. Descriptive statistics to summarise drug concentrations in plasma and DBS in the whole sample set, and separated to determine any significant difference between drug concentrations in those with HBV viral suppression vs non-viral suppression (persistent or breakthrough viraemia).
2. Univariable and multivariable analysis to identify associations between drug concentrations and demographic, clinical and laboratory parameters.

Based on a study in the UK[^1^](https://paperpile.com/c/3htKyC/eIgj), and pilot data from South Africa (unpublished data), after receiving treatment for a year, we anticipate 75% of the population to have viraemic suppression on treatment and 25% to have detectable viraemia (non-suppressed).

Assuming we measure TFV-DP concentrations from 200 DBS samples (150 suppressed, 50 non-suppressed), with a mean concentration of 1000 fmol/punch (with SD 400), we would have 80% power, at a two-sided α of 0.05, to detect a difference of approximately 180 fmol/punch between groups. There is no agreed therapeutic threshold for tenofovir in the context of HBV treatment, these assumptions are based on prior data generated in studies of HIV[^2,3^](https://paperpile.com/c/3htKyC/SKL6+Sd2Z), and our own preliminary data which will be expanded through an expert consortium[^4^](https://paperpile.com/c/3htKyC/wO1H).

##### 5.2 HBV Whole Genome Sequencing (WGS)

We will focus HBV-specific methods on new plasma or serum samples (from adults living with or without HIV coinfection), identified through Phases I and II of EVOLVE-HBV, using the published ‘HEP-TILE’ method (based on a tiled amplicon enrichment followed by sequencing using Oxford Nanopore Technology)[^5^](https://paperpile.com/c/3htKyC/cJ6A5).

Samples from individuals with HIV/HBV coinfection recruited through Vukuzazi are also available at the University of Oxford, UK. Nucleic acid has already been extracted from these samples, and residual sample (that would otherwise be returned to the Vukuzazi programme), may be used to inform and optimize laboratory and bioinformatic methods for co-sequencing of HBV to improve sensitivity of detection and to provide proof-of-principle for approaches that can sequence both HIV and HBV, as well as other organisms in the sample. Methods optimisation may also include use of metagenomic approaches which derive reads for any organisms that are present in blood.

Analysis of pathogen genetic data cannot be formally powered *a priori*, because sample diversity is unknown, and the success of the sequencing protocol depends on the viral loads in material collected (which for HBV infection are typically low[^6^](https://paperpile.com/c/3htKyC/0iOu)).

Analysis will follow a stepwise approach, involving first routine quality control and metrics of sequence data. We will determine genotype/subgenotype based on use of reference sequences[^7^](https://paperpile.com/c/3htKyC/zdm1). In the determination of polymorphisms potentially associated with drug resistance, we will first draw on an existing catalogue of relevant sequence changes[^8^](https://paperpile.com/c/3htKyC/ICB7), and subsequently consider the potential for a sequence-agnostic approach to identifying new polymorphisms which may affect outcomes of infection or its treatment. Where relevant, analysis will be supported by adjustment for multiple testing (control family-wise error rate (FWER) and false-discovery rate (FDR) with p-value adjustment).

Analysis protocols will be shared in the public domain, e.g. on [protocols.io](http://protocols.io).

Analysis of HBV whole genome sequence data is shown in **Supplementary Table 2.**

###### Supplementary Table 2: Analysis approaches for HBV sequence data

| **Sequencing output*** | **Metrics and/or analysis** |
| --- | --- |
| Quality metrics of sequence data | - Number of HBV reads, % genome coverage, depth. |
| HBV genotypes and subgenotypes | - Analysis based on a published panel of reference sequences (for example[^7^](https://paperpile.com/c/3htKyC/zdm1)). |
| HBV recombination, dual infection, coinfection | - Evidence of HBV recombination; - Evidence of dual infection (with different HBV genotypes/subgenotypes; - Co-infection (with HIV and/or other hepatitis viruses, and/or other endemic viruses if metagenomic approaches are used). |
| Phylogenetic reconstruction | - Analysis of transmission networks; - Molecular epidemiology of HBV sequence data in study population compared to other regional / national / international data. |
| HBV sequence polymorphisms | - Prevalence of polymorphisms (including sequence changes associated with drug resistance, vaccine escape, and disease outcomes such as hepatocellular carcinoma, for example based on existing publications and catalogues[^8,40^](https://paperpile.com/c/3htKyC/xsDi+ICB7)); - Relationships between sequence changes (insertions / deletions / polymorphisms) and clinical and laboratory biomarkers of disease progression such as VL and ALT; - Covariation between polymorphisms; - Relationship between HBV sequence changes and other demographic and clinical variables collected in the population data set. |

ALT: alanine aminotransferase; HBV: hepatitis B virus; HIV: human immunodeficiency virus; VL: viral load. Next generation sequencing proposed using a ‘HEPTILE’ protocol which generates whole genome sequences using pre-amplification by PCR followed by Oxford Nanopore Technology sequencing[^5^](https://paperpile.com/c/3htKyC/cJ6A5).

##### 5.3 ALT point of care testing (POCT)

The study aims to evaluate the role of ALT lateral flow testing using POCT, (e.g. BioPoint® antigen immunoassay; Nanjing DeShi Diagnostic Technology Co. Ltd)[^9^](https://paperpile.com/c/3htKyC/oWQiX). We will test 200 samples in the Vukuzazi stored/retrospective sample set (stratified to include a range of ALT values) using an ALT POCT. We will also test 100 samples in the prospectively recruited population (aiming to split between 50 HBV+ and 50 HBV-). This will give us a view of implementation in practice. The POCT results will be compared against quantitative laboratory results from NHLS which are generated through a validated diagnostic facility.

Concordance between the POCT and quantitative laboratory assay will be determined to assess the sensitivity and specificity of the POCT for a given ALT threshold, based on clinical guidelines (typically regarding ALT upper limit of normal (ULN) as 40 U/L in the first instance, but with the potential to amend this threshold if POCT thresholds are lowered, for example in accordance with HBV treatment guidelines which specify more stringent ALT thresholds).

The prevalence of an elevated ALT>ULN in this population will be estimated based on Phase I data.

The POCT will be done in an AHRI clinical/laboratory space by a trained member of staff. Feedback will be collated from lab teams undertaking the test regarding ease of use and readability to inform further development and/or scale up of testing. The result will be recorded (positive/negative/equivocal) along with the option to record qualitative notes on test kit use and performance. The result will not inform any clinical action, as the test is not yet validated.

We aim to report a primary endpoint of sensitivity and/or specificity with 95% confidence intervals. Positive predictive value and negative predictive value will be reported as secondary or exploratory endpoints. As an example, if we test 200 samples in a setting where 20% of the population has ALT >ULN, with an expected POCT sensitivity to detect ALT >ULN of 0.9 (95% CI 76.3 - 97.2), and expected specificity 75% (95% CI 67.6 - 81.5), Positive Predictive Value is 47.4% (95% CI 40.3 - 54.5) and Negative Predictive Value is 96.8% (95% CI 92.2% to 98.7%).

##### 5.4 Host genetics

Host genetics, particularly killer-cell immunoglobulin-like receptors (KIR) and human leukocyte antigen (HLA), have been associated with HBV persistence, susceptibility, protection, vaccine, treatment response and HIV infection control[^10–13^](https://paperpile.com/c/3htKyC/VpOzi+DkxGW+C4DUH+CvjZN); however, such data for Africa are sparse[^13–23^](https://paperpile.com/c/3htKyC/CvjZN+FhgXn+IqiPu+mMrV6+1qhsX+xEGuH+Qh1Le+ujFU9+SlmNX+s97Jx+RocuO). T cell exhaustion is part of the immunological phenotype of HBV persistence[^24^](https://paperpile.com/c/3htKyC/EQUxs), and both CD4+ and CD8+ T-cells play roles in determining outcome[^25^](https://paperpile.com/c/3htKyC/v09v7).

An external company will, independently or collaboratively, perform exome and genome wide analyses (ExWAS and GWAS, respectively) and phenome-wide association analyses to identify novel associations between genetic variants and all phenotypic traits, across all diseases, traits and health outcomes available of DNA samples from the Vukuzazi programme, under the terms of existing governance approvals, making host genetic data (including HLA I and HLA II genotypes) available for analysis in the EVOLVE project. They may, in collaboration or independently, perform meta-analyses and control matching with other cohorts, as needed. They may perform, in collaboration or independently, epidemiological (phenotype only) analyses that will be used to describe the phenotypes of the participants in the cohorts and their associations.

Prospectively, any new study participants in EVOLVE-HBV will be provided with written and verbal information and asked for their informed consent for us to generate and analyse genetic data (matching the terms of the Vukuzazi protocol). For participants who wish to opt out of genetic testing, this will be recorded, and we will not generate these data but they are still eligible for participation in other aspects of the research.

EVOLVE-HBV is not powered to assess the relationship between host genetics and disease outcomes, but by contributing anonymised host genetic data and supporting study metadata to pooled analysis, we can contribute to larger studies with the aim of understanding the role of genetic polymorphisms in HBV disease outcomes in different populations. However, where numbers allow, we will describe or examine the association between HLA alleles and HBV VL, HBV genotype, and liver function tests using multiple linear regression models adjusted for confounding factors. To evaluate the association of HLA genotypes with HBV disease control, conditional logistic regression will be used to estimate odds ratios (ORs) and 95% confidence intervals (CIs). The outcome of interest will be disease progression, defined using conventional clinical and laboratory markers.

##### 5.5 Humoral immune responses

Human neutralising antibodies against HBV surface antigen (HBsAg) mainly target its extracellular antigenic loop or major hydrophilic region, and also target the Pre-S1 and Pre-S2 domains. Individuals who are vaccinated or achieve HBsAg clearance (‘functional cure’) develop neutralising antibodies that target shared epitopes in HBsAg[^26^](https://paperpile.com/c/3htKyC/sYn8w). People exposed to HBV infection (whether with HBsAg-positive status or following HBsAg clearance) typically test positive for non-neutralising HBV core antibodies.

Frozen aliquots of plasma/serum will be accessed for measuring HBV antibody levels and their effector functions in different population groups to account for both HIV and HBV infection (**Supplementary table 3)**.

###### Supplementary Table 3: Aims for studies of the humoral immune response to HBV

| ***Primary aims:*** | (i) To measure the magnitude of IgG responses (total IgG and IgG subclasses) to Hepatitis B surface antigen.  (ii) To determine associations between the magnitude of antibody responses and disease biomarkers, particularly viral load, in people living with HBV (‘HBV Cases’). |
| --- | --- |
| ***Secondary aim:*** | To measure antibody-mediated Fc effector functions. |
| ***Extension aims:*** | To undertake neutralisation assays *in vitro.* |

Humoral immune responses will be measured in :

‘HBV Cases’: **n=160** HBsAg-positive samples (anti-HBs-negative)

‘HBV-immune controls’: **n=160** matched HBsAg-negative samples, divided into two groups:

(i) Uninfected, vaccinated (anti-HBs-positive, anti-HBc-negative); **n=80**

(ii) Previously infected and cleared HBsAg (anti-HBs-positive, anti-HBc-positive); **n=80**

‘HBV-negative controls’: uninfected, unvaccinated (anti-HBs-negative, anti-HBc-negative); **n=10**

In each group, we aim to represent equal numbers of males and females and include People Living With HIV (PLWH) and HIV-uninfected individuals. We will aim to consider HBsAg proteins by genotype, with consideration of genotype D as the most widely available laboratory assay, and genotype A as the most prevalent in the South African population.

**Methods for Primary Aim**:

Quantitation of total anti-HBs IgG will be derived at NHLS laboratories using a validated diagnostic assay platform.

The magnitude of anti-HBs IgG1-IgG4 antibody subclasses will be measured in serum using a multiplex ELISA. Magnetic microsphere beads coupled with HBsAg will coat Millipore high-binding luminex plates overnight. Diluted serum will be added and incubated for 2 hours at room temperature. The plates will be washed with PBS-Tween1%. The Horseradish Peroxidase (HRP)-conjugated anti-human IgG1-IgG4 antibodies will be added for the detection of antibody responses. Anti-human IgG1-IgG4 levels will be compared between the HBsAg-positive and HBsAg-negative participants.

**Methods for Secondary Aim:**

The ability of serum antibodies to initiate cellular cytotoxity or phagocytosis will be measured by flow cytometry. High throughput antibody functional assays were previously set-up at AHRI and HIV Pathogenesis Program (HPP). Diluted serum samples will be added in a 96-well plate, coated with HBsAg. This will be followed by either freshly isolated natural killer cells from a healthy individual or macrophage cell line (THP1 cells). Surface and intracellular staining will be performed, followed by the data acquisition on the BD LSR Fortessa cytometer or an equivalent platform.

***Extension aims:***

Subject to additional funding and work with external collaborators, we will undertake neutralization assays. For example, HBV will be produced by HepDE19 cells in the absence of tetracycline as previously described[^27^](https://paperpile.com/c/3htKyC/g6ZR1), and neutralisation activity will be measured in HepG2 NTCP-expressing cells using an immunofluorescence assay. The cell nucleus will be stained with DAPI, viewed under the fluorescent microscope and images will be obtained. The percentage of cells positive for HBc (HBc+) cells will be calculated by normalizing to the virus-only control, which will be used as a reference.

##### 5.6 Occult HBV Infection (OBI)

OBI is defined as the presence of detectable HBV DNA in the absence of detectable HBsAg. We will retain up to 1000-1500 samples for OBI screening from Phase I.

DNA extraction will be undertaken using a validated nucleic acid extraction kit according to the manufacturer’s instructions. Samples will be tested for HBV DNA using an RT-PCR approach, in batches, with controls that are confirmed to be positive and negative for HBV-DNA. The testing will be replicated for validation purposes at NHLS laboratories (Abbott Alinity quantitative HBV assay) with a lower limit of quantitation (LLoQ) of 10 IU/mL (1.0 Log IU/mL) and upper limit of quantitation (ULoQ) 1,000,000,000 IU/mL (9.0 Log IU/mL).

The OBI analysis in this study is exploratory given that the prevalence in this setting is unknown. Based on existing data, OBI prevalence in SA is highly variable, estimated between 5%-23%, depending on the study population[^28–32^](https://paperpile.com/c/3htKyC/uZYXE+bkqSg+RzydN+M6Ev3+4HRkf). With 1500 samples, if true prevalence is 23%, the precision is ±2% (21-25%); for 1000 samples, precision is ±2.3%; with 500 samples precision is ±4%.

Participants whose samples have tested positive for OBI (HBsAg-negative, HBV DNA positive) will be contacted in order to inform them of their status. This knowledge is important because we can advise that they should not donate blood/tissue for transfusion or transplantation. There is potential reactivation risk, especially in patients commencing immunosuppressive drugs, so knowledge of their status in case of future treatment is also of benefit to the individual to inform their healthcare team.

Investigating for OBI is not current standard clinical practice, and OBI is not a group for which hepatocellular carcinoma (HCC) screening is currently recommended. However, results from this work can inform future public health strategy.

##### 5.7 HCV and HDV coinfection

Estimating the prevalence of Hepatitis C VIrus (HCV) and Hepatitis D Virus (HDV) in this population are secondary / extension aims. The prevalence of HCV infection in this population is likely to be low based on previous research in Southern Africa (<1% overall, most of which is concentrated in high-risk populations such as people who inject drugs)[^33,34^](https://paperpile.com/c/3htKyC/4buI7+pv0BR). HDV prevalence is low in South Africa (e.g. 0.05% in existing reports)[^35,36^](https://paperpile.com/c/3htKyC/BnEc2+bWbZ9). On these grounds, confidence around point estimates is likely to be wide, and we have not included HDV screening as part of our primary analysis due to cost constraints. However, this screening may be undertaken subject to securing additional resources.

At the time of developing this protocol, access to Direct-acting Antiviral Agents (DAAs) for HCV in this setting in South Africa would require a Section 21 application. There is local experience of treating some patients in Durban from a donation, as well as documented success in HCV treatment elsewhere in SA, e.g. with 12 weeks of sofosbuvir and daclatasvir[^33,37^](https://paperpile.com/c/3htKyC/4buI7+3Uvi). However, specialist consultation will be offered for any person found to be living with HCV infection. Treatment would be conducted according to clinical guidelines (outside the remit of this study), with sustained virological response (SVR) confirmed by undetectable HCV RNA at least 12 weeks after the end of treatment.

#### **Appendix 6: Data dictionary**

The data dictionary contains all information to be collected by the study. Domains will either be completed by a member of the study team (clinician / study nurse / researcher), in collaboration with the laboratory team supporting assimilation of results from the NHLS laboratory, or through linkage with other local databases (**Supplementary table 4)**. A REDCap database (https://project-redcap.org/) has been established with support from the experienced AHRI data management team.

###### Supplementary Table 4: Data dictionary for EVOLVE-HBV study

| **Category** | | **Details to be collected** |
| --- | --- | --- |
| 1.0 Demographics | 1.1 | Unique study number for EVOLVE-HBV study |
|  | 1.2 | Vukuzazi identifier number |
|  | 1.2 | Sex at birth (male/female/prefer not to say/not recorded) |
|  | 1.3 | Year of birth |
|  | 1.4 | Area of residence (link to data stored in Geographical Information System, GIS) |
|  | 1.5 | Ethnicity (use local classification) |
| 2.0 Data from NHLS laboratory serosurvey | 2.1 | HBsAg status (positive/negative/equivocal/not done) |
|  | 2.2 | Anti-HBs status (positive/negative/equivocal/not done) |
|  | 2.3 | Anti-HBs antibody titre, positive defined as >=10 IU/ml (positive/negative/equivocal/not done)  Record quantitative value |
|  | 2.4 | Anti-HBc status (positive/negative/equivocal) |
|  | 2.5 | HBV VL (for select samples including all that test HBsAg-positive and a subset screened for occult infection) |
|  | 2.6 | HBeAg status (positive/negative/equivocal) for all those testing HBsAg positive |
|  | 2.7 | ALT (quantitative value) |
|  | 2.8 | HDV RNA positive/negative/not tested |
|  | 2.9 | HDV RNA quantitation for those testing HDV RNA positive |
| 3.0 Clinical history and assessment (data collected at the time of participant review in a clinical environment, study Phase II) | 3.1 | Date of clinic review |
|  | 3.2 | Confirm informed consent for participation (yes/no) and check/complete details in section 1 (demographics)  (if no, then no further data to be collected) |
|  | 3.3 | Was this participant included in the Phase I serosurvey? (yes/no/don't know) |
|  | 3.4 | HBV status (HBsAg status) based on current information at the time they are reviewed/recruited (positive/negative/equivocal/not known) |
|  | 3.5 | HIV status based on current information at the time they are reviewed/recruited (positive/negative/equivocal/not known) – with date of diagnosis of positive |
|  | 3.5 | Pregnancy status (pregnant/not pregnant/not known)  If pregnant, do not proceed, if not pregnant or unknown then can proceed) |
|  | 3.6 | Weight in kg |
|  | 3.7 | Height in cm |
|  | 3.8 | Waist circumference in cm |
|  | 3.9 | Hip circumference in cm |
|  | 3.10 | Blood pressure systolic (mmHg) |
|  | 3.11 | Blood pressure diastolic (mmHg) |
|  | 3.12 | Existing diagnosis of diabetes (yes/no) with date of diagnosis if known |
|  | 3.13 | Existing chronic or current diagnoses with dates (free text box e.g. renal disease, hypertension, cardiac disease) |
|  | 3.14 | Clinical evidence of liver disease (yes/no for each)   - Jaundice - Hepatomegaly - Ascites - Oedema - Spider naevi - Weight loss, nausea, anorexia - Other features (free text at the discretion of the clinical team) |
|  | 3.15 | Personal history suggestive of liver disease (e.g. based on features listed in 2.4 or a clinical diagnosis) |
|  | 3.16 | Family history suggestive of liver disease, including liver cancer (e.g. based on features listed in 2.4, or a clinical diagnosis) |
|  | 3.17 | Fibroscan data   - Scan operator - Date of study - Probe size (M / XL) - Fibrosis score (kPa) - CAP score (dB/m) - Quality metrics of scan (IQR) |
|  | 3.18 | Details of HIV infection and treatment from TIER.Net system for people living with HIV infection (date of ART initiation, viral load results and dates, CD4 count results and dates, type of ART regimen, and dates of clinic appointments) |
| 4.0 Questionnaire data | 4.1 | Occupation |
|  | 4.2 | Do you currently drink alcohol (yes/no)  How many days a week do you drink alcohol (tick box scale)  How many drinks do you consume in a day when you are drinking? (tick box scale)  Have you ever struggled with alcohol so that it affects your work or relationships? (yes/no) |
|  | 4.3 | Do you have a personal history of liver disease? (yes/no/uncertain) |
|  | 4.4 | Has anyone in your family ever had liver disease? (yes/no/uncertain) |
|  | 4.5 | Do you smoke? (current/past/never)  If smoker, state number of cigarettes per day |
|  | 4.6 | Do you use any herbal medicine/muthi? (currently using/sometimes use/occasional use/never use)  If yes to herbal medicine, please provide details (Herbal type / name) |
|  | 4.7 | Does your diet include ground nuts/peanuts/peanut oil? (often/infrequently/rarely/never/not sure)  Does your diet include maize? (often/infrequently/rarely/never/not sure) |
|  | 4.8 | Have you ever lived in a region where you were in regular contact with lake water? (yes/no)  If yes to lake water contact, where? |
|  | 4.9 | If you had liver disease or liver infection would you be willing to see a local doctor or nurse 2-3 times a year for routine check? (yes/no/uncertain) |
|  | 4.10 | If you had liver disease or liver infection would you be willing to go to a district hospital 2-3 times a year for routine check? Eg Hlabisa (yes/no/uncertain) |
|  | 4.11 | If you had liver disease or liver infection would you be willing to go to a regional hospital 2-3 times a year for routine check? Eg Durban (yes/no/uncertain) |
|  | 4.12 | If you had liver disease or liver infection would you be willing to take daily tablets long term to reduce the risk of complications? (yes/no/uncertain) |
|  | 4.13 | If you had liver disease and you needed routine check-up, which of the following would cause problems for you to attend (list of options – please tick all that apply) |
|  | 4.14 | If you had hepatitis B infection how would you feel about telling your close family members (in the same household)? (list of options provided) |
|  | 4.15 | If you had hepatitis B infection how would you feel about telling your friends and colleagues? (list of options provided) |
|  | 4.16 | How do you feel hepatitis B is perceived in this community? (list of options provided) |
|  | 4.17 | To what extent do you agree with the following - people living with HBV infection might feel shame/stigma/discrimination? (list of options provided) |
|  | 4.18 | Do you know that hepatitis B infection is linked to the development of liver cancer? Yes/maybe/no |
|  | 4.19 | Medication – tick box for medications prescribed for HIV, TB, diabetes, blood pressure (with free text box for other medications) |
| 5.0 Conclusion of clinical review | 5.1 | Information on hepatitis B and liver disease provided to participant (select from drop down list of which any number can be ticked, oral information / written information / online information) |
|  | 5.2 | Case flagged for clinical review by study team (yes review needed / no review not needed) |
|  | 5.3 | Outcome of clinical review – dropdown options (reviewed and no action needed, reviewed and further action needed), free text box to state what actions recommended |
| 6.0 Clinical Laboratory data generated from samples collected on face to face (prospective) review | 6.1 | Hepatitis B surface antigen |
|  | 6.2 | Hepatitis B core antibody |
|  | 6.3 | Hepatitis B surface antibody |
|  | 6.4 | Hepatitis B e antigen |
|  | 6.5 | Hepatitis B DNA viral load (including cases of occult infection) |
|  | 6.6 | HIV viral load (if applicable) |
|  | 6.7 | CD4+ T cell count and % (if applicable) |
|  | 6.8 | ALT |
|  | 6.9 | AST |
|  | 6.10 | GGT |
|  | 6.11 | ALP |
|  | 6.12 | BR |
|  | 6.13 | Alb |
|  | 6.14 | Creatinine |
|  | 6.15 | Platelet count |
|  | 6.16 | AFP |
|  | 6.17 | Urinary electrolytes |
|  | 6.18 | Urinary protein:creat ratio |
|  | 6.19 | Liver fibrosis scores based on lab data (GPR), |
|  | 6.20 | Liver fibrosis scores based on lab data (APRI) |
|  | 6.21 | Liver fibrosis scores based on lab data (Fib-4) |
|  | 6.22 | Co-infection: HCV (yes/no/equivocal/not done) |
|  | 6.23 | Co-infection: HDV (yes/no/equivocal/not done) |
|  | 6.24 | Co-infection: HIV (yes/no/equivocal/not done) |
| 7.0 Research laboratory data | 7.1 | HBV sequence data and metrics (coverage, depth, genotype/subgenotype, recombination, polymorphisms, ± coinfection with other viruses identified as a result of metagenomic sequencing). |
|  | 7.2 | Tenofovir concentration (sample type, assay used, date of assay, quantitation of free drug or drug metabolite). |
|  | 7.3 | In vitro characterisation of antibody quantity and activity (titres, avidity, breadth etc). |
|  | 7.4 | Host genetic data (either generated previously through participation in the Vukuzazi programme, or generated de novo from samples collected in this study). |
| 8.0 Social sciences data | 8.1 | Qualitative data based on community discussions and interviews. |
|  | 8.2 | Quantitative data regarding beliefs, behaviours, barriers to care. |

EVOLVE-HBV: Evaluation of Vukuzazi LiVEr disease - Hepatitis B; HBsAg: hepatitis B surface antigen; anti-HBs: hepatitis B surface antibodies; anti-HBc: hepatitis B core antibodies; HBV: hepatitis B virus; HBeAg: hepatitis B e antigen; ALT: alanine aminotransferase; HDV: hepatitis D virus; RNA: ribonucleic acid; HIV: human immunodeficiency; ART: antiretroviral therapy; TB: tuberculosis; cm: centimeter; mmHg: millimeters of mercury; DNA: deoxyribonucleic acid; AST: Aspartate aminotransferase; GGT: gamma-glutamyltansferase; ALP: alkaline phosphatase; BR: bilirubin; Alb: albumin; AFP: alpha fetoprotein; GPR: GGT to platelets ratio; APRI: AST to Platelet Ratio Index; FIB-4: Fibrosis-4 Index; HCV: hepatitis C virus; DP: diphosphate (metabolite of Tenofovir disoproxil fumarate); AHRI: Africa Health Research Institute

#### **Appendix 7: Supplementary details of data management plan**

##### 7.1 Data storage

Based on AHRI Data Classification Standard, all personally identifiable information are regarded as sensitive data and are stored in a separate table in the database with access restricted only to the Research Data Manager (not be included in the analytical datasets archived on the AHRI data repository). If access to this data is required for any research purpose, this is authorised on a case-by-case basis by the data controllers.

Data will be made available on the AHRI data enclave per AHRI Data Access Policy (<https://www.ahri.org/wp-content/uploads/2023/10/AHRI-PAIA-Manual-v2.1.pdf>). The data enclave is a virtual environment launched from the researcher's own desktop but operating on a remote server under AHRI control. This platform provides restricted access, preventing users from downloading, emailing, copying, or moving files. Outputs are vetted by a member of the research data management team, and if analytical results contain no personally identifiable information the results can be made available for download from the data repository.

Although confidentiality cannot be guaranteed, every effort is made to ensure that all clinical records are protected. All forms, reports, and other records are identified in a manner designed to maintain confidentiality, and kept in a secure storage area with limited access provided to trained staff only. Data for groups of fewer than five individuals will not be shared to avoid the possibility of individuals being recognised.

##### 7.2 Data availability

Once data have been stored in the study database, they become the responsibility of the head of research data management, who assesses and manages data quality. Research data management administers a data repository that contains all datasets required for analysis for the study. They ensure that the repository has access control measures that enforce the data access criteria determined by the study data steering committee. Access to the datasets is granted to those who meet minimum requirements set out by the study data steering committee.

Data sharing between AHRI and other study sites is done using secure methods of transfer, subject to the terms of the study contract, and in compliance with local and international data regulation laws e.g. SA’s Protection of Personal Information (POPI) Act and European Union’s General Data Protection Regulation (GDPR).

At the end of the study, anonymised analytical datasets will be created, documented and archived on the AHRI data repository (<https://data.ahri.org/index.php/home>) with a digital object identifier (DOI). All direct identifiers will be removed, and indirect identifiers will be minimised or aggregated to reduce re-identification risk in accordance with institutional data protection guidelines. Research metadata will be publicly available in fully anonymised form to support manuscripts that are generated from the study. De-identified datasets and data analysis code links will be provided upon manuscripts acceptance.

#### **Appendix 8: Exemplar sample size calculation for clinical data**

We considered the anticipated power to detect an association between an abnormal biomarker (e.g. based on liver function tests with alanine transferase elevated above the upper limit of normal) and HBV infection. Anticipated power based on two controls recruited for each case is shown in **Supplementary Table 5.**

###### Supplementary Table 5: Anticipated power for detection of varied odds ratios (OR), according to the prevalence of an abnormal biomarker and varying numbers of HBV cases.

| Prevalence of abnormal biomarker in controls | Number of HBV cases | Number of HBV negative controls | OR threshold | | |
| --- | --- | --- | --- | --- | --- |
|  |  |  | 2.25 | 2.50 | 3.00 |
| 5% | 100 | 200 | 44% | 55% | 74% |
| 10% | 100 | 200 | 67% | 80% | 93% |
| 15% | 100 | 200 | 80% | 89% | 97% |
| 5% | 150 | 300 | 60% | 73% | 89% |
| 10% | 150 | 300 | 84% | 92% | 99% |
| 15% | 150 | 300 | 92% | 97% | >99% |
| 5% | 200 | 400 | 72% | 84% | 96% |
| 10% | 200 | 400 | 92% | 97% | >99% |
| 15% | 200 | 400 | 97% | 99% | >99% |

OR: odds ratio; HBV: hepatitis B virus

#### **Supplementary References**

1.	[Wang, T. *et al.* Distinct virologic trajectories in chronic hepatitis B identify heterogeneity in response to nucleos(t)ide analogue therapy. *JHEP Rep.* **7**, 101229 (2025).](http://paperpile.com/b/3htKyC/eIgj)

2.	[Anderson, P. L. *et al.* Intracellular tenofovir-diphosphate and emtricitabine-triphosphate in dried blood spots following directly observed therapy. *Antimicrob. Agents Chemother.* **62**, (2018).](http://paperpile.com/b/3htKyC/SKL6)

3.	[Castillo-Mancilla, J. R. *et al.* Tenofovir, emtricitabine, and tenofovir diphosphate in dried blood spots for determining recent and cumulative drug exposure. *AIDS Res. Hum. Retroviruses* **29**, 384–390 (2013).](http://paperpile.com/b/3htKyC/Sd2Z)

4.	[Matthews, P. *et al.* Therapeutic drug monitoring for hepatitis B virus infection (’TDM 4 HBV'). Preprint at https://doi.org/](http://paperpile.com/b/3htKyC/wO1H)[10.6084/M9.FIGSHARE.C.8095186](http://dx.doi.org/10.6084/M9.FIGSHARE.C.8095186) [(2025).](http://paperpile.com/b/3htKyC/wO1H)

5.	[Lumley, S. F. *et al.* Whole genome sequencing of hepatitis B virus using tiled amplicon (HEPTILE) and probe based enrichment on Illumina and Nanopore platforms. *Sci Rep* **15**, 5795 (2025).](http://paperpile.com/b/3htKyC/cJ6A5)

6.	[Downs, L. O. *et al.* Bimodal distribution and set point HBV DNA viral loads in chronic infection: retrospective analysis of cohorts from the UK and South Africa. *Wellcome Open Res* **5**, 113 (2020).](http://paperpile.com/b/3htKyC/0iOu)

7.	[McNaughton, A. L., Revill, P. A., Littlejohn, M., Matthews, P. C. & Ansari, M. A. Analysis of genomic-length HBV sequences to determine genotype and subgenotype reference sequences. *J. Gen. Virol.* (2020) doi:](http://paperpile.com/b/3htKyC/zdm1)[10.1099/jgv.0.001387](http://dx.doi.org/10.1099/jgv.0.001387)[.](http://paperpile.com/b/3htKyC/zdm1)

8.	[Mokaya, J. *et al.* Hepatitis B virus resistance to tenofovir: fact or fiction? A systematic literature review and structural analysis of drug resistance mechanisms. *Wellcome Open Res.* **5**, 151 (2020).](http://paperpile.com/b/3htKyC/ICB7)

9.	[Howell, J. *et al.* Validation of a novel point-of-care test for alanine aminotransferase measurement: A pilot cohort study. *Liver Int* **43**, 989–999 (2023).](http://paperpile.com/b/3htKyC/oWQiX)

10.	[Terme, M., Ullrich, E., Delahaye, N. F., Chaput, N. & Zitvogel, L. Natural killer cell-directed therapies: moving from unexpected results to successful strategies. *Nat Immunol* **9**, 486–494 (2008).](http://paperpile.com/b/3htKyC/VpOzi)

11.	[Bashirova, A. A., Thomas, R. & Carrington, M. HLA/KIR restraint of HIV: surviving the fittest. *Annu Rev Immunol* **29**, 295–317 (2011).](http://paperpile.com/b/3htKyC/DkxGW)

12.	[Hu, Z. *et al.* New loci associated with chronic hepatitis B virus infection in Han Chinese. *Nat Genet* **45**, 1499–1503 (2013).](http://paperpile.com/b/3htKyC/C4DUH)

13.	[Kamatani, Y. *et al.* A genome-wide association study identifies variants in the HLA-DP locus associated with chronic hepatitis B in Asians. *Nat Genet* **41**, 591–595 (2009).](http://paperpile.com/b/3htKyC/CvjZN)

14.	[Kibar, F. *et al.* Role of KIR genes and genotypes in susceptibility to or protection against hepatitis B virus infection in a Turkish cohort. *Med Sci Monit* **20**, 28–34 (2014).](http://paperpile.com/b/3htKyC/FhgXn)

15.	[Mardian, Y. *et al.* Genetic polymorphisms of HLA-DP and isolated anti-HBc are important subsets of occult hepatitis B infection in Indonesian blood donors: a case-control study. *Virol J* **14**, 201 (2017).](http://paperpile.com/b/3htKyC/IqiPu)

16.	[Matthews, P. C. *et al.* HLA-A is a Predictor of Hepatitis B e Antigen Status in HIV-Positive African Adults. *J Infect Dis* **213**, 1248–1252 (2016).](http://paperpile.com/b/3htKyC/mMrV6)

17.	[Shah-Hosseini, A. *et al.* The impact of KIR-HLA genotype on hepatitis B virus clearance in Iranian infected individuals. *Med Microbiol Immunol* **206**, 463–470 (2017).](http://paperpile.com/b/3htKyC/1qhsX)

18.	[Thio, C. L. *et al.* Class II HLA alleles and hepatitis B virus persistence in African Americans. *J Infect Dis* **179**, 1004–1006 (1999).](http://paperpile.com/b/3htKyC/xEGuH)

19.	[Thursz, M. R. *et al.* Association between an MHC class II allele and clearance of hepatitis B virus in the Gambia. *N Engl J Med* **332**, 1065–1069 (1995).](http://paperpile.com/b/3htKyC/Qh1Le)

20.	[Varbanova, V. *et al.* KIR/HLA ligands immunogenetics markers associated with outcome of hepatitis B virus infection in the Bulgarian population. *Biomed Pap Med Fac Univ Palacky Olomouc Czech Repub* **165**, 270–276 (2021).](http://paperpile.com/b/3htKyC/ujFU9)

21.	[Wang, T. *et al.* Human leukocyte antigen class I association with occult hepatitis B virus infection in the Shaanxi Han group: Analysis at the haplotype level. *J Gene Med* **24**, e3393 (2022).](http://paperpile.com/b/3htKyC/SlmNX)

22.	[Wang, T. *et al.* High resolution HLA-DRB1 analysis and shared molecular amino acid signature of DRβ1 molecules in Occult hepatitis B infection. *BMC Immunol* **23**, 22 (2022).](http://paperpile.com/b/3htKyC/s97Jx)

23.	[Zhang, Q. *et al.* HLA-DP polymorphisms affect the outcomes of chronic hepatitis B virus infections, possibly through interacting with viral mutations. *J Virol* **87**, 12176–12186 (2013).](http://paperpile.com/b/3htKyC/RocuO)

24.	[Peeridogaheh, H. *et al.* Current concepts on immunopathogenesis of hepatitis B virus infection. *Virus Res* **245**, 29–43 (2018).](http://paperpile.com/b/3htKyC/EQUxs)

25.	[Zhang, W. *et al.* Hepatitis B Virus-Specific Cellular Immunity Contributes to the Outcome of Occult Hepatitis B Virus Infection. *Front Microbiol* **13**, 850665 (2022).](http://paperpile.com/b/3htKyC/v09v7)

26.	[Wang, Q. *et al.* A Combination of Human Broadly Neutralizing Antibodies against Hepatitis B Virus HBsAg with Distinct Epitopes Suppresses Escape Mutations. *Cell Host Microbe* **28**, 335–349.e6 (2020).](http://paperpile.com/b/3htKyC/sYn8w)

27.	[Cai, X. *et al.* A virus-like particle of the hepatitis B virus preS antigen elicits robust neutralizing antibodies and T cell responses in mice. *Antiviral Res* **149**, 48–57 (2018).](http://paperpile.com/b/3htKyC/g6ZR1)

28.	[Bell, T. G., Makondo, E., Martinson, N. A. & Kramvis, A. Hepatitis B virus infection in human immunodeficiency virus infected southern African adults: occult or overt--that is the question. *PLoS One* **7**, e45750 (2012).](http://paperpile.com/b/3htKyC/uZYXE)

29.	[Lukhwareni, A., Burnett, R. J., Selabe, S. G., Mzileni, M. O. & Mphahlele, M. J. Increased detection of HBV DNA in HBsAg-positive and HBsAg-negative South African HIV/AIDS patients enrolling for highly active antiretroviral therapy at a Tertiary Hospital. *J Med Virol* **81**, 406–412 (2009).](http://paperpile.com/b/3htKyC/bkqSg)

30.	[Amponsah-Dacosta, E., Selabe, S. G. & Mphahlele, M. J. Evolution of the serologic and virologic course of occult HBV infection in therapy experienced HIV co-infected patients. *J Med Virol* **90**, 291–303 (2018).](http://paperpile.com/b/3htKyC/RzydN)

31.	[Sondlane, T. H. *et al.* High prevalence of active and occult hepatitis B virus infections in healthcare workers from two provinces of South Africa. *Vaccine* **34**, 3835–3839 (2016).](http://paperpile.com/b/3htKyC/M6Ev3)

32.	[Firnhaber, C. *et al.* Prevalence of hepatitis B virus (HBV) co-infection in HBV serologically-negative South African HIV patients and retrospective evaluation of the clinical course of mono- and co-infection. *Int J Infect Dis* **16**, e268–72 (2012).](http://paperpile.com/b/3htKyC/4HRkf)

33.	[Sonderup, M. W. *et al.* Direct-acting antiviral therapy for hepatitis C: The initial experience of the University of Cape Town/Groote Schuur Hospital Liver Clinic, South Africa. *S Afr Med J* **110**, 112–117 (2020).](http://paperpile.com/b/3htKyC/4buI7)

34.	[Sonderup, M. W. *et al.* Expanding the epidemiological understanding of hepatitis C in South Africa: Perspectives from a patient cohort in a rural town. *S Afr Med J* **111**, 783–788 (2021).](http://paperpile.com/b/3htKyC/pv0BR)

35.	[Matthews, P. C. *et al.* Prevalence and Characteristics of Hepatitis B Virus (HBV) Coinfection among HIV-Positive Women in South Africa and Botswana. *PLoS One* **10**, e0134037 (2015).](http://paperpile.com/b/3htKyC/BnEc2)

36.	[Andersson, M. I. *et al.* The epidemiology of hepatitis B virus infection in HIV-infected and HIV-uninfected pregnant women in the Western Cape, South Africa. *Vaccine* **31**, 5579–5584 (2013).](http://paperpile.com/b/3htKyC/bWbZ9)

37.	[Saayman, E., Hechter, V. B., Sishwili, N. S. & Sonderup, M. W. A decentralized point of service triple prevention and treatment model for hepatitis B, C and HIV in people who inject drugs in South Africa. *Int. J. Drug Policy* **143**, 104894 (2025).](http://paperpile.com/b/3htKyC/3Uvi)

38.	[World Health Organization. *Guidelines for the Prevention, Diagnosis, Care and Treatment for People with Chronic Hepatitis B Infection*. (World Health Organization, 2024).](http://paperpile.com/b/3htKyC/wpwZj)

39.	[Petroff, D. *et al.* Assessment of hepatic steatosis by controlled attenuation parameter using the M and XL probes: an individual patient data meta-analysis. *Lancet Gastroenterol. Hepatol.* **6**, 185–198 (2021).](http://paperpile.com/b/3htKyC/GWX4b)

40.	[Maponga, T. G. *et al.* A putative hepatitis B virus sequence motif associated with hepatocellular carcinoma in South African adults. *Ann. Hepatol.* 101763 (2025).](http://paperpile.com/b/3htKyC/xsDi)
